# A global view of gene expression in lithium and zinc treated sea urchin embryos: new components of gene regulatory networks

**DOI:** 10.1186/gb-2007-8-5-r85

**Published:** 2007-05-16

**Authors:** Albert J Poustka, Alexander Kühn, Detlef Groth, Vesna Weise, Shunsuke Yaguchi, Robert D Burke, Ralf Herwig, Hans Lehrach, Georgia Panopoulou

**Affiliations:** 1 Max-Planck Institut für Molekulare Genetik, Evolution and Development Group, Ihnestrasse 73, 14195 Berlin, Germany; 2 University of Victoria, Departments of Biology and Biochemistry/Microbiology, 3800 Finnerty Road, Victoria, British Columbia, Canada V8P 5C5; 3 US National Institutes of Health, National Institute of Dental and Craniofacial Research, 30 Convent Drive, MSC 4326, Bethesda. Maryland 20815, USA

## Abstract

**Background:**

The genome of the sea urchin *Strongylocentrotus purpuratus *has recently been sequenced because it is a major model system for the study of gene regulatory networks. Embryonic expression patterns for most genes are unknown, however.

**Results:**

Using large-scale screens on arrays carrying 50% to 70% of all genes, we identified novel territory-specific markers. Our strategy was based on computational selection of genes that are differentially expressed in lithium-treated embryos, which form excess endomesoderm, and in zinc-treated embryos, in which endomesoderm specification is blocked. Whole-mount *in situ *hybridization (WISH) analysis of 700 genes indicates that the apical organ region is eliminated in lithium-treated embryos. Conversely, apical and specifically neural markers are expressed more broadly in zinc-treated embryos, whereas endomesoderm signaling is severely reduced. Strikingly, the number of serotonergic neurons is amplified by at least tenfold in zinc-treated embryos. WISH analysis further indicates that there is crosstalk between the Wnt (wingless int), Notch, and fibroblast growth factor signaling pathways in secondary mesoderm cell specification and differentiation, similar to signaling cascades that function during development of presomitic mesoderm in mouse embryogenesis. We provide differential expression data for more than 4,000 genes and WISH patterns of more than 250 genes, and more than 2,400 annotated WISH images.

**Conclusion:**

Our work provides tissue-specific expression patterns for a large fraction of the sea urchin genes that have not yet been included in existing regulatory networks and await functional integration. Furthermore, we noted neuron-inducing activity of zinc on embryonic development; this is the first observation of such activity in any organism.

## Abstract 2

Novel territory-specific markers from the sea urchin *Strongylocentrotus purpuratus *have been identified using screens for genes that are differentially expressed in lithium-treated embryos, which form an excess of endomesoderm, and in zinc-treated embryos, in which endomesoderm specification is blocked.

## Background

Body plan development is controlled by large gene regulatory networks (GRNs). Such networks consist of components that accurately specify cell fate at defined times during development via their physical interaction, or in the case of transcription factors via their binding to *cis*-regulatory DNA elements. One of the best studied developmental GRNs is the sea urchin endomesoderm GRN, which includes almost 50 genes [1,2]. These genes were uncovered in part through three array screens: a subtractive screen, in which RNA from lithium-treated embryos was subtracted with RNA isolated from cadherin injected embryos [3]; a *Brachyury *target gene screen [4]; and a screen for pigment cell-specific genes [5]. Comparison of the endoderm network between vertebrates (mouse, *xenopus*, and zebrafish) showed that many components have been conserved. Common key zygotic factors are the Nodal-related transforming growth factor-β ligands, the Mixlike (paired box) family of homeodomain transcription factors, the *Gata4*/*Gata5*/*Gata6 *zinc-finger transcription factors and the HMG box transcription factor *Sox17 *[6-10]. Orthologs of some of these genes are components of the sea urchin endomesoderm GRN. Examples include *SpGataE *and *SpGataC *(orthologs of *Gata4*/*Gata5*/*Gata6 *and *Gata1*/*Gata2*/*Gata3*, respectively), *SpFoxA *(ortholog of *FoxA1 *[*HNF3b*], which in *Xenopus *is a target of *Mixer*), and *SpOtx *(ortholog of *Otx2*, which in *Xenopus *is induced by *Sox17*). However, comparison of the vertebrate and sea urchin endomesoderm network also reveals that many sea urchin orthologs of vertebrate endomesoderm genes are absent from the respective sea urchin GRN.

This could be due to the fact that the existing sea urchin endomesoderm GRN is built progressively, starting from genes found to be regulated in the initial screens; this raises the possibility that nodes of the endomesoderm network that are not affected by the above subtractive hybridizations have not yet been explored. In addition, some genes employed in the sea urchin endomesoderm GRN are apparently absent from vertebrate endomesoderm GRNs. The aim of this study is to identify additional genes that are associated with developmental patterning, primarily focusing on endomesoderm specific genes but also on genes that are involved in ectoderm differentiation and patterning. We then add these genes to the existing GRNs or create novel GRNs that describe sea urchin embryonic development.

The early sea urchin embryo develops two primary axes: the animal-vegetal axis and the oral-aboral axis. Most of the endodermal and mesodermal cells are derived from the vegetal half, whereas the animal cells contribute to neural and non-neural ectodermal territories. During gastrulation the ectoderm is divided into an oral side, which flattens and is the site where the mouth secondarily breaks through, and a rounded aboral side, which is seperated by the ciliary band region.

Activation of the sea urchin endomesoderm GRN is initiated at the molecular level as a result of nuclearization of β-catenin initially in the vegetal micromeres (at the fourth cleavage) and subsequently in the macromeres and their progenitor blastomeres veg2 and part of veg1. The nuclearization of β-catenin in the micromeres at the 16-cell stage is also the earliest molecular evidence of an animal-vegetal axis in *Strongylocentrotus purpuratus *[11-14].

**Figure 1:**
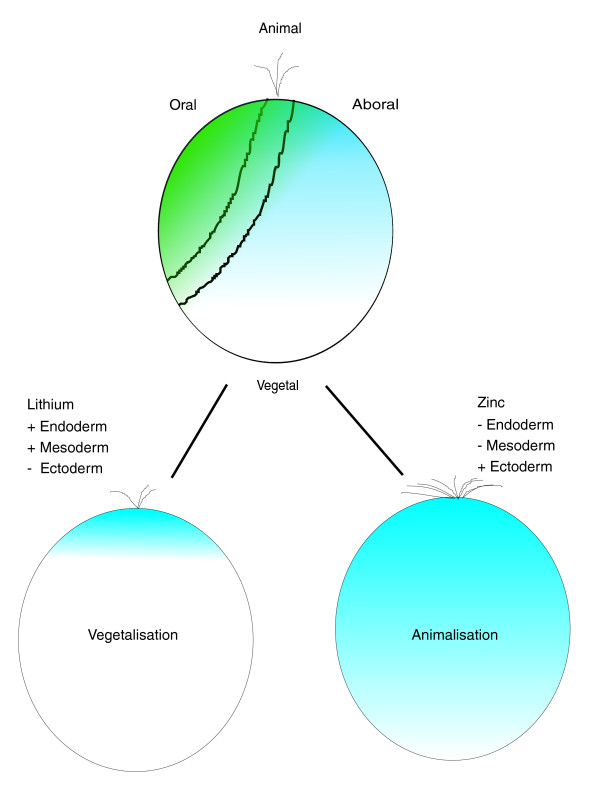
Normal development and perturbations. Normal sea urchin embryos (top) develop two primary axis: the animal-vegetal axis and the oral-aboral axis. Nuclearization of β-catenin in cells on the vegetal side initiates endomesoderm specification. Later on the ectoderm is divided into an oral and aboral side, which is comparable to the dorso-ventral axis in vertebrates. Treating embryos with lithium chloride leads to enhanced nuclearization of β-catenin and, as a result, a shift in cell fate toward vegetal and formation of excess endomesoderm (left). Conversely zinc sulfate treatment prevents endomesoderm formation (right). The molecular basis for zinc sulfate action is unknown, as is the effect of these drugs on the ectoderm.

Reagents exist for manipulation of the GRNs that specify the embryonic axis. Lithium chloride acts as a vegetalizing (posteriorizing) agent by directly binding glycogen synthase kinase-3β, thus freeing up β-catenin, which then enters the nucleus and activates target genes via a complex with Tcf/Lef [14] (Figure 1 shows a sketch of the resulting axis perturbations). As result of the vegetalization, the endomesodermal domain is expanded at the expense of ectodermal territories. A recent study suggested that lithium chloride treatment induces an increase in endoderm at the expense of the ectoderm, but without alterating the mesodermal territories, because the expression domain of *Frizzled5*/*8 *at the animal pole is eliminated whereas its expression at the secondary mesenchyme cells (SMCs) is not affected [15]. Furthermore, recent evidence based on study of *Nodal *suggests that lithium chloride also intervenes with the oral-aboral axis of the embryo, because the region expressing the oral marker *Nodal *is reduced and shifted to the animal side [16], which is consistent with the conversion of part of the ectoderm to endoderm. Oral-aboral axis is established before the sixth cleavage and is dependant on signals from the vegetal pole [16,17]. Complementary to lithium treatment, zinc treatment animalizes (anteriorizes) the embryos and leads to embryos with no or reduced endomesodermal cells [18-20].

Using these reagents we conducted separate array hybridizations of lithium chloride or zinc sulfate treated and normal embryos. Because lithium vegetalizes and zinc complementarily animalizes embryos, we would expect endomesoderm-specific genes to be upregulated in embryos treated with lithium and downregulated in embryos treated with zinc sulfate, whereas ectoderm-specific genes should exhibit the opposite pattern.

Hybridizations were carried out on nonredundant arrays that correspond to 50% to 70% of all sea urchin genes [21]. In our experimental design we have used repetitions of experiments in order to calculate sensitivity as a factor of reproducibility. We deliberately did not amplify or subtract any probes, because these procedures run the risk for distorting the representation of different sequences in the RNA sample. In addition, they can interfere with the identification of (for instance, they may remove) highly expressed genes, which can also be territory specific markers. Differentially expressed genes were analyzed by whole-mount *in situ *hybridization (WISH) from early blastula stages (10 hours) to the pluteus stage (90 hours) during normal embryonic development, and certain identified marker genes were also analyzed for expression in treated embryos. In this way we identified key molecules of endomesoderm and oral-aboral axis differentiation, novel territories, and new highly dynamic expression patterns in the sea urchin embryo. A total of about 700 out of more than 4.000 differentially expressed genes representing all functional protein classes have thus far been analyzed by WISH. All WISHs were annotated and deposited in a database that is freely accessible [22]. The differential expression data are available in the Array Screens Database [23]. As the screens progress, this database will continue to be expanded.

## Results

### Strategy for expression profiling

We generated a robust strategy for profiling the expression of genes differentially expressed during early development in sea urchin. We compared the conditions of embryos vegetalized by lithium treatment (excess endomesoderm) and animalized by zinc treatment (excess ectodermal territories). Expression profiles were established for embryos at different developmental stages. They were established for the midblastula stage at 20 hours after fertilization for lithium-vegetalized embryos and at a midgastrula stage for zinc treatment (38 hours; see Materials and methods, below, for treatment details). We decided to analyze the expression profile of a midgastrula stage of development (38 hours) for the animalized embryos because it is at this stage that a first phenotypic effect becomes visible (a thickened animal plate and the absence of gut structures). In addition, the use of a later stage is also useful for establishing an expression profile catalog throughout development (Poustka AJ, unpublished data).

For expression profiling experiments to be valid, they must exhibit good sensitivity and reproducibility; hence in order to identify significantly regulated genes, it is necessary to generate enough data points to allow reliable statistical analyses to be conducted.

RNA was isolated from embryos subjected to treatment and control embryos simultaneously, and was hybridized simultaneously on 12 array copies in order to prevent differences resulting from discrepant handling procedures. For each probe, six different filter copies were hybridized (for each experiment) to collect 24 data points per clone (each clone is spotted in duplicate). This high number of repetitions enables the calculation of reproducibility values based on the coefficient of variation of the replicate signal intensities for each cDNA clone. The statistical tests (Student's *t*-test 1, Welch test, Wilcoxon test, and a permutation-based test) were calculated for all clones. A total of 3,456 copies of an *Arabidopsis *clone were used to adapt the *P *values, ensuring that an experimental false-positive rate of 5% is not exceeded (for details, see Herwig and coworkers [24]).

In order to minimize measurement error resulting from cross-talk between neighboring spots, we made two different arrays for each set of clones with two different spotting patterns, both of which are used in each experiment. Arrays were made on nylon filters carrying polymerase chain reaction (PCR) amplification products of the inserts of 35,238 cDNA clones, representing about 20,000 genes of the sea urchin *S. purpuratus*. This set of clones was selected as a low-redundancy set, as indicated by normalization by oligonucleotide fingerprinting and expressed sequence tag (EST) analysis [21]. A re-evaluation with the now available draft of the sea urchin genome sequence verifies that the established gene catalog contains a tag for more than 50% of all sea urchin genes. Out of a total of 28,944 predicted sea urchin gene models (Glean3) [25], 14,638 do not match an EST sequence, which would mean that almost 50% of the gene predictions are already covered by an EST. Of the 27,217 EST clusters, however, 10,698 do reversely not match any Glean3 gene prediction. This indicates that, as expected, untranslated region sequences are not properly predicted in the Glean3 gene set and hence that the number of sea urchin genes tagged in our EST catalog is well over 50%.

### Lithium-zinc *in silico *subtraction and performance evaluation

A total of 6,581 clones were identified as being differentially expressed, according to the criteria described in the Materials and methods (below; all data are available at the sea urchin embryo WISH database [23]). We estimate that these clones represent about 4,000 different genes, based on comparison with the gene predictions (Glean3) of the recently completed sea urchin genome sequence [25]. Because lithium vegetalizes and zinc complementarily animalizes embryos, we would expect endomesoderm-specific genes to be upregulated in lithium-treated embryos and simultaneously downregulated in zinc-treated embryos, whereas ectoderm-specific genes should exhibit the opposite pattern. We selected 81 clones that are upregulated in the hybridizations with lithium chloride-treated embryos and downregulated in the hybridizations with zinc sulfate-treated embryos (referred to hereafter as 'LiUpZiDown' clones) and 151 LiDownZiUp clones, of which 39 and 101 clones, respectively, were analyzed by WISH. Whereas the percentage of these clones giving restricted expression patterns was very high (61% and 68% for LiUpZiDown and LiDownZiUp, respectively), the localization results were striking. Of the clones predicted to be localized to the endomesoderm domain from the LiUpZiDown fraction, 96% were indeed localized to an endomesodermal domain during embryogenesis. Likewise, only 19% of the LiDownZiUp group localized to an endomesodermal domain, whereas the rest were expressed in an ectodermal domain.

As the next step, we evaluated the quality of all of the results by examining the differentially expressed genes by quantitative real-time PCR (Q-PCR). Statistical analysis (see above) should ensure that the false-positive rate stays below 5%. The high number of repetitions and the resulting statistical evaluation gave us the confidence to select even marginally regulated clones, such as those exhibiting a minimal expression change of 1.3 and a significant reproducibility value (*P *value) of minimally e^-3 ^from the set of all regulations. We selected genes of good (*P *< e^-5^), medium (*P *= e^-4 ^to e^-3^), and poor (*P *> e^-2^) e values (the last being below the 5% quantile for significantly regulated clones; see Materials and methods, below). Tables 1, 2, and 3 summarize the values from the array and the Q-PCR experiments for 71 genes.

**Table 1:** Differential expression data based on array experiments and Q-PCR of endomesoderm marker genes

EM markers	Expression	Regulation	*P *value	Q-PCR ratio ± error
Brachyury 537REA_5B8	EM	Li 1.69 Zn 0.42	1.08 × e^-06^ 3.89 × e^-03^	1.64 ± 0.21 0.33 ± 0.10
Blimp1 537REA_16B13	E	Li 1.33 Zn 0.42	3.98 × e^-02 ^ 2.58 × e^-03^	1.78 ± 0.09 0.53 ± 0.04
CoA-reductase 536REAsu2_4L15	EM	Li 0.40 Zn 1.31	5.68 × e^-08 ^ 1.20 × e^-03^	0.44 ± 0.03 1.71 ± 0.50
Delta 536REAsu4_17C2	SMC	Li 1.79 Zn NA	1.64 × e^-01 ^ NA	4.86 ± 1.05 0.95 ± 0.14
Dlx 537REA_9O11	EM-Oect	Li 0.09 Zn NA	2.60 × e^-05 ^ NA	0.03 ± 0.00 0.58 ± 0.26
Endo16 536REAsu2_5N1	E	Li 1.20 Zn 0.46	3.00 × e^-01 ^ 2.05 × e^-04^	1.76 ± 0.34 1.70 ± 0.12^a^
Eve 537REA_2G8	E	Li 1.33 Zn nd	4.89 × e^-03 ^ nd	0.93 ± 0.01^a ^ 1.73 ± 0.10
FoxA 537REA_10P13	EM+Oect	Li 1.37 Zn na	1.37 × e^-06 ^ na	3.18 ± 0.12 1.69 ± 0.43
GataE 537REA_3C9	E	Li 1.55 Zn 0.48	2.77 × e^-03 ^ 1.71 × e^-03^	3.06 ± 0.92 0.23 ± 0.04
Hex SpSMBLAS_124N22	M	Li 1.54 Zn nd	3.99 × e^-01 ^ nd	3.22 ± 0.53 0.33 ± 0.03
Hox11/13b 537REA_12K1	E	Li 1.69 Zn na	3.44 × e^-09 ^ na	na na
KRL PMC_BG781437	EM	Li Zn		1.90 ± 0.03 5.95 ± 0.66
Lox 536REAsu4_13M18	E	Li ne Zn 0.36	ne 5.17 × e^-03^	ne 0.04 ± 0.01
Notch RUDIREA_30E15	E	Li 1.27 Zn na	4.84 × e^-01 ^ na	1.81 ± 0.18 13.5 ± 3.41
P19 537REA_15K13	PMC	Li 1.07 Zn 1.58	7.82 × e^-01 ^ 3.43 × e^-03^	1.66 ± 0.33 2.14 ± 0.22
PMAR1	PMC	Li na Zn na	na na	1.77 ± 0.29 0.50 ± 0.02
Prox RUDIREA_15N17	M	Li 0.57 Zn na	2.40 × e^-04 ^ na	0.81 ± 0.16 1.46 ± 0.27
Six3 RUDIREA_40B23	Apical, later +EM	Li 0.72 Zn 0.10	7.83 × e^-02 ^ 6.46 × e^-03^	0.39 ± 0.04 1.15 ± 0.15^a^
SM50 RUDIREA_5L2	PMC	Li 0.57 Zn 1.55	1.09 × e^-05 ^ 1.20 × e^-04^	1.07 ± 0.19^a ^ 18.12 ± 2.93
SMAD2 RUDIREA_24O7	PMC	Li 0.50 Zn NA	2.49 × e^-01 ^ NA	0.52 ± 0.09 1.20 ± 0.32
Snail RUDIREA_13L18	EM	Li 0.99 Zn 0.26	9.72 × e^-01 ^ 9.05 × e^-05^	0.24 ± 0.01 0.09 ± 0.01
Sox4 RUDIREA_2H10	EM, later +apical	Li 1.15 Zn nd	4.23 × e^-01 ^ nd	1.67 ± 0.21 1.05 ± 0.08
SuH 621REA_14C17	EM	Li 0.24 Zn NA	3.75 × e^-02 ^ NA	4.24 ± 0.95^a ^ 4.61 ± 1.50
T-Brain 621Rea_6N24	M	Li 0.82 Zn na	6.93 × e^-01 ^ na	1.40 ± 0.31^a ^ 2.18 ± 0.12
Tbx6 RUDIREA_29D1	M	Li 0.64 Zn 0.74	6.71 × e^-05 ^ 1.78 × e^-05^	0.33 ± 0.15 0.16 ± 0.04
Unknown 536REAsu4_13G12	EM	Li 2.23 Zn 4.05	2.69 × e^-07 ^ 5.52 × e^-10^	4,68 ± 0.72 11,01 ± 0,27
Wnt3 RUDIREA_28M14	EM	Li 0.89 Zn NA	7.86 × e^-01 ^ NA	2.29 ± 0.97^a ^ 0.09 ± 0.03
Wnt5 RUDIREA_16P23	EM	Li 1.25 Zn NA	1.97 × e^-01 ^ NA	6.31 ± 2.31 1.08 ± 0.08
Wnt8 537REA_10K11	EM	Li 1.35 Zn NA	4.19 × e^-03 ^ NA	2.49 ± 0.07 11.36 ± 1.13

The first column gives the gene name and the clone ID, both of which can be used to query the described database [22] for additional data. In the second column the localization of expression in the embryo is given, where EM is endomesoderm, E is endoderm, M is mesoderm, PMC is primary mesenchyme cell, and SMC is secondary mesenchyme cell, Oect is oral ectoderm. The third column (Regulation) gives the differential expression ratios (expression in treatment/expression control) based on the array experiment for lithium (Li) and zinc (Zn) treated embryos (values above 1 indicate upregulation and values below 1 indicate downregulation). The column '*P *value' indicates the statistical probability that the regulation could happen by chance (see Materials and methods for detail). The column Q-PCR (quantitative real-time polymerase chain reaction) gives the differential expression ratios (expression in treatment/expression control) and the error, as determined by Q-PCR. (Values expressed in copies of mRNA molecules/embryo are provided via the expressed sequence tag database [75]; see Materials and methods for details on Q-PCR). ^a^Differential expression based on array and Q-PCR data do not correlate. na, not analyzed; nd, no statistically relevant differential expression; ne, not expressed; ?, expression pattern unknown.

**Table 2:** Differential expression data based on array experiments and Q-PCR of ectoderm marker genes

Ectoderm markers	Expression	Regulation	*P *value	Q-PCR ratio ± error
Bmp2/4 SpSMBLIT_68K21	Oral	Li 1.32 Zn 1.54	3.13 × e^-01 ^ 7.33 × e^-01^	1.27 ± 0.29 0.97 ± 0.25
Chordin 537REA_13L23	Oral	Li nd Zn 0.40	nd 4.55 × e^-05^	1.19 ± 0,19 0.41 ± 0,12
Goosecoid RUDIREA_9C8	Oral	Li 1.05 Zn 0.27	8.43 × e^-01 ^ 4.13 × e^-03^	0.75 ± 0.08^a ^ 0.16 ± 0.06
Lefty 536REAsu4_7H9	Oral	Li 1.80 Zn nd	1.91 × e^-07 ^ nd	0.98 ± 0.22^a ^ 2.17 ± 0,14
Nodal 536REA_98I13	Oral	Li na Zn na	na na	1.34 ± 0,11 4.12 ± 0,23
IrxA SpSMBLAS_51E6	Aboral	Li 0.38 Zn	2.51 × e^-09^	0.08 ± 0.05 1.60 ± 0.02
Nkx2.2 536REAsu4_10G11	Aboral	Li 0.37 Zn nd	7.69 × e^-08 ^ nd	0.15 ± 0.13 5.87 ± 0.15
Spec2A	Aboral	Li nd Zn nd	nd nd	na 10.64 ± 1.67
Tbx2 RUDIREA_26D12	Aboral	Li 0.20 Zn	3.50 × e^-12^	0.12 ± 0.01 1.42 ± 0.24
Dp-Hbn SpSMBLAS_141N1	Apical	Li 0.14 Zn nd	1.41 × e^-07 ^ nd	0.05 ± 0.02 1.03 ± 0.21
FoxJ RUDIREA_13I13	Apical	Li 1.40 Zn 2.29	2.86 × e^-02 ^ 3.26 × e^-07^	0.93 ± 0.20^a ^ 1.84 ± 0.04
FoxQ 537REA_3F18	Apical	Li 0.24 Zn 4.51	3.55 × e^-14 ^ 5.01 × e^-07^	0.15 ± 0.03 4.66 ± 0.78
Glass 536REAsu2_5I1	Apical	Li 0.52 Zn na	2.99 × e^-01 ^ na	ne 0.03± 0.01
ZFhpf4 537REA_15C23	Apical	Li 0.24 Zn na	7.78 × e^-07 ^ na	1.20 ± 0.00 2.05 ± 0.15
Hypothetical RUDIREA_15C22	Apical + SMC late	Li 0.59 Zn 4.27	4.79 × e^-01 ^ 3.24 × e^-02^	0.20 ± 0.06 1.11 ± 0.10
Mox SpSMBLAS_131A20	Apical, serotonergic	Li 0.49 Zn nd	8.23 × e^-02 ^ nd	1.76 ± 0.22^a ^ 1.39 ± 0.21
Radical spoke protein RUDIREA_39F2	Apical	Li 1.56 Zn na	5.75 × e^-04 ^ na	1.14 ± 0.10 2.11 ± 0.45
sFRP1/5 536REAsu4_11O4	Apical	Li 0.42 Zn 1.99	7.10 × e^-04 ^ 3.67 × e^-04^	0.11 ± 0.04 2.14 ± 0.08
Hairy1 537REA_10P14	Cilliary band +?E	Li 0.77 Zn	5.78 × e^-06^	0.62 ± 0.04 1.54 ± 0.25
onecut 538REA_9E3	Cilliary band	Li 0.40 Zn 0.74	3.20 × e^-04 ^ 5.88 × e^-03^	0.68 ± 0.06 1.84 ± 0.24^a^
Pax2 RUDIREA_22J20	Cilliary band	Li 1.14 Zn na	7.71 × e^-01 ^ na	3.72 ± 0.50 0.11 ± 0.02
AEX3 RUDIREA_5J10	Entire ectoderm, off vegetal	Li 0.64 Zn 5.34	1.37 × e^-08 ^ 3.83 × e^-12^	0.78 ± 0.26 9.11 ± 0.90
Hatching enzyme 538REA_2G05	Entire ectoderm	Li 3,39 Zn 4.35	4.42 × e^-07 ^ 1.25 × e^-03^	7.55 ± 0.55 11.42 ± 0.87
Otx 537REA_12D12	Entire ectoderm	Li 0.65 Zn na	1.01 × e^-04 ^ na	0.96 ± 0.09 2.43 ± 0.14
Soxb1 RUDIREA_25A17	Entire ectoderm	Li 1.28 Zn nd	9.93 × e^-02 ^ nd	0.87 ± 0.05^a ^ 1.43 ± 0.27
Soxb2 536REAsu4_4A13	Entire ectoderm	Li 1.44 Zn	6.71 × e^-05^	0.79 ± 0.12^a ^ 1.12 ± 0.27
SpAN RUDIREA_29D20	Entire ectoderm	Li 2.19 Zn nd	7.72 × e^-07 ^ nd	1.88 ± 0.13 1.73 ± 0.14

The first column gives the gene name and the clone ID, both of which can be used to query the described database [22] for additional data. In the second column the localization of expression in the embryo is given, where E is endoderm and SMC is secondary mesenchyme cell. The third column (Regulation) gives the differential expression ratios (expression in treatment/expression control) based on the array experiment for lithium (Li) and zinc (Zn) treated embryos (values above 1 indicate upregulation and values below 1 indicate downregulation). The column '*P *value' indicates the statistical probability that the regulation could happen by chance (see Materials and methods for detail). The column Q-PCR (quantitative real-time polymerase chain reaction) gives the differential expression ratios (expression in treatment/expression control) and the error, as determined by Q-PCR. (Values expressed in copies of mRNA molecules/embryo are provided via the expressed sequence tag database [75]; see Materials and methods for details on Q-PCR). ^a^Differential expression based on array and Q-PCR data do not correlate. na, not analyzed; nd, no statistically relevant differential expression; ne, not expressed; ?, expression pattern unknown.

**Table 3:** Differential expression data based on array experiments and Q-PCR of genes with unknown or ubiquitous expression pattern

Other genes	Expression	Regulation	P Value	Q-PCR ratio
Arginine kinase 536REAsu4_15B20	?	Li 2.20 Zn na	7.29 × e^-07 ^ na	1.95 ± 0.15 2.07 ± 0.47
ß-catenin 538REA_2O22 RUDIREA_22E13	Ubiquitous	Li 0.39 Zn na	4.77 × e^-03 ^ na	0.85 ± 0.11 0.79 ± 0.16
SpZ12	?	Li 1.44 Zn 2.29	2.55 × e^-01 ^ 2.05 × e^-02^	2.03 ± 0.37 2.66 ± 0.40
Hmx, NkX5.1 537REA_6C02	?	Li 0.20 Zn 1.86	1.18 × e^-14 ^ 5.87 × e^-03^	0.05 ± 0.03 1.20 ± 0.32
Tcf/Lef 536REAsu4_11P24	?	Li 1.37 Zn na	7.88 × e^-02 ^ na	0.73 ± 0.13^a ^ 1.42 ± 0.29
WntA RUDIREA_33L4	?	Li 1.07 Zn nd	8.13 × e^-01 ^ nd	0.39 ± 0.13^a ^ 0.24 ± 0.12
Wnt1	?	Li na Zn na	na na	ne ne
Wnt4 536REAsu4_6C19	?	Li 1.09 Zn NA	6.69 × e^-01 ^ NA	1.66 ± 0.36 1.73 ± 0.21
Wnt6	?	Li na Zn na	na na	1.55 ± 0.19 0.24 ± 0.01
Wnt7	?	Li na Zn na	na na	ne 0.03 ± 0.02
Wnt9	?	Li na Zn na	na na	ne 0.29 ± 0.03
Wnt10	?	Li na Zn na	na na	1.43 ± 0.29 1.30 ± 0.14
Wnt16	?	Li na Zn na	na na	2.79 ± 0.81^a ^ 0.84 ± 0.12

The first column gives the gene name and the clone ID, both of which can be used to query the described database [22] for additional data. In the second column the localization of expression in the embryo is given. The third column (Regulation) gives the differential expression ratios (expression in treatment/expression control) based on the array experiment for lithium (Li) and zinc (Zn) treated embryos (values above 1 indicate upregulation and values below 1 indicate downregulation). The column '*P *value' indicates the statistical probability that the regulation could happen by chance (see Materials and methods for detail). The column Q-PCR (quantitative real-time polymerase chain reaction) gives the differential expression ratios (expression in treatment/expression control) and the error, as determined by Q-PCR. (Values expressed in copies of mRNA molecules/embryo are provided via the expressed sequence tag database [75]; see Materials and methods for details on Q-PCR). ^a^Differential expression based on array and Q-PCR data do not correlate. na, not analyzed; nd, no statistically relevant differential expression; ne, not expressed; ?, expression pattern unknown.

Overall, we generated and compared differential expression data for 80 regulations (namely zinc or lithium) between array and Q-PCR data. In 17 cases the regulations were not in agreement, indicating an experimental false positive rate of 21% for the entire set of 6,581 differentially regulated clones (indicated by ^'a' ^in Tables 1, 2, and 3).

To identify the biologic pathways affected by the treatments, we analyzed the expression data in terms of pathways. To sort sea urchin genes into pathways we mapped the ESTs of the regulated clones on our arrays to the predicted sea urchin genes (Glean3) of the recently sequenced genome [25] and then searched to determine whether their human orthologs are involved in pathways listed in the Kyoto Encyclopedia of Genes and Genomes pathway database [26]. The results indicate a statistically significant differential regulation of the mitogen-activated protein kinase and transforming growth factor-β pathway in zinc-treated embryos.

### Expression profile with lithium chloride treatment

**Figure 2:**
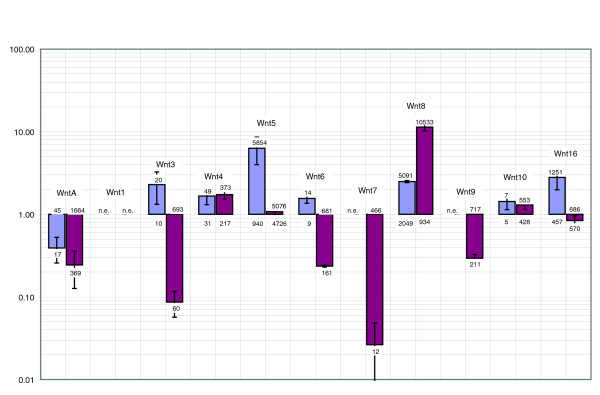
Expression of Wnt genes in lithium and zinc treated embryos. Quantitative real-time polymerase chain reaction (Q-PCR) analysis of all wingless int (Wnt) genes of the sea urchin *Strongylocentrotus purpuratus*. Measurements were done at blastula stage (20 hours) for lithium-treated embryos (purple bars) and gastrula stage (38 hours) for zinc-treated embryos (pink bars). Data are presented in a logarithmic style. Bars above 1 indicate upregulation and bars below 1 indicate downregulation. The numbers given on top or bottom of bars are the number of mRNA molecules/embryo in normal or treated embryos, respectively. For instance, the number of transcripts for *wntA *is 45 in normal 20 hours embryos and 17 in lithium-treated embryos (blue bar), and the number of transcripts of *wnt5 *is 940 in normal 20 hours embryos and 5,854 in lithium-treated 20 hours embryos. Where n.e. (not expressed) is indicated the gene is not expressed at this stage at all, either in control or in treated embryos. Also see Tables 1 and 3 and the text for further detail.

We then assessed the efficacy of the lithium chloride treatment through examining the behavior of known sea urchin endomesoderm genes in the above hybridizations. As expected, we found that endomesoderm-specific genes (such as *Brachyury*, *gata-e*, *foxa*, *hox11/13b*, *notch*, *wnt8 *[1,3], *krl *[27] and *endo16 *[28]), which are central components of the endomesoderm GRN, are all upregulated with the exception of *eve*, which we found not to be significantly regulated (as verified by Q-PCR; Table 1). Because lithium treatment is thought to activate Wnt (wingless int) signaling by stabilizing β-catenin, we investigated the expression of Wnt genes in treated embryos. A Q-PCR survey of all 11 Wnt genes in *S. purpuratus *reveals that Wnts 5, 8, and 16 are expressed (> 100 copies/per embryo) at 20 hours of development (which is the time point at which lithium chloride measurements were obtained). Furthermore, all three are significantly upregulated in lithium-treated embryos, indicating and confirming a strong positive response to lithium treatment of Wnt signaling (see Figure 2 for Wnt gene Q-PCR findings, and Tables 1 and 3).

**Figure 3:**
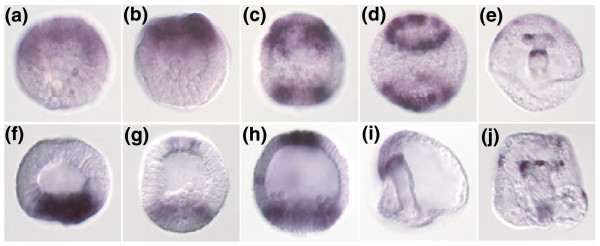
Opposing expression patterns of *six3 *and *sox4*. Whole-mount *in situ *hybridization (WISH) analysis of the developmental expression pattern of the transcription factors *six3 *and *sox4*. **(a to e) ***six3*; **(f to j) ***sox4*. The animal side is located to the top in all images. *Six3 *expression starts as early as 8 hours of development (8 hours embryo in panel a and 10 hours in panel b) at the animal side of the embryo. At the mesenchyme blastula stage (20 hours in panel c and flattened embryo in panel d), the animal expression clears from the central apical plate (apical organ) and at the same time forms a ring-like expression around the vegetal pole as well. In the pluteus (panel e) expression is detectable in a part of one coelomic pouch and at the forgut-midgut constriction. In contrast, *sox4 *is initially expressed on the vegetal side (14 hours embryo in panel f). Starting from 18 hours (panel g, and 20 hours in panel h) of development, expression also starts in the apical plate. At gastrula stage (panel i) expression is detected at the archenteron tip, and in the pluteus (panel j) expression can be detected in various secondary mesoderm cell derivatives, including some coelomic pouch cells.

**Figure 4:**
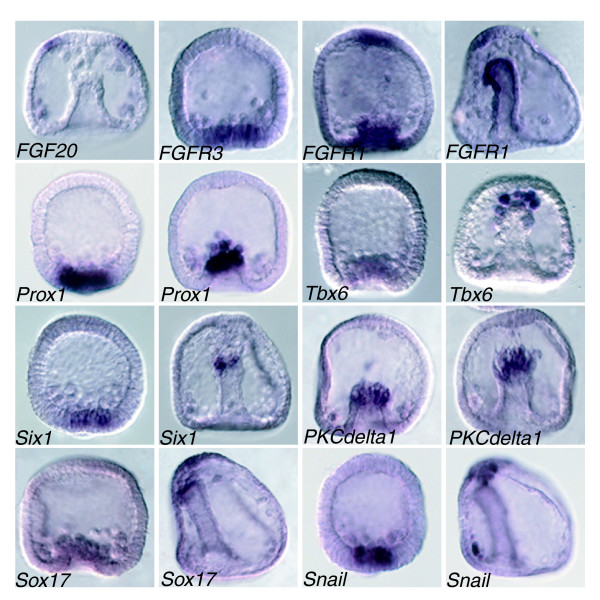
Coexpression of genes in SMC cells. Whole-mount *in situ *hybridization (WISH) analysis of examples of signaling and transcription factor genes identified in this screen. *FGF20 *(*Sp-FGF9*/16/20), the only fibroblast growth factor present in the sea urchin genome, is expressed in primary mesenchyme cells (PMCs) and around the apical organ during gastrulation, whereas two receptors identified in this screen are expressed in adjacent secondary mesenchyme cells (SMCs; *FGFR3*, blastula stage) and in SMCs and the central apical region (*FGFR1*, left blastula, right gastrula). The transcription factors *Prox1*, *Tbx6*, *Six1*, *Sox17*, and *snail *are expressed in SMCs during gastrulation, as is a *PKCdelta1 *gene. In all pictures the animal sides of the embryos is located towards the top. Annotated images of additional stages can be found in the WISH database [22].

**Figure 5:**
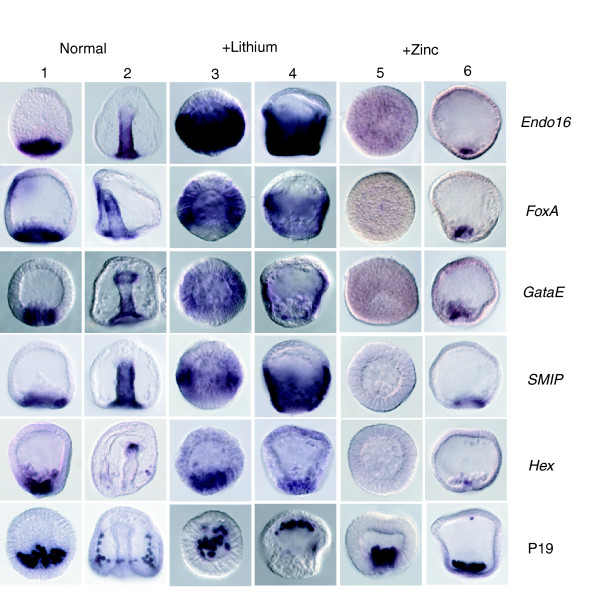
Expression of endomesoderm markers in normal, lithium-treated and zinc-treated embryos. Shown are whole-mount *in situ *hybridizations (WISHs) of endomesodermal marker genes on blastula stage (columns 1, 3, and 5) and gastrula stage (columns 2, 4, and 6) sea urchin embryos. The genes under considerations are indicated on the right hand side. *Endo16*, *FoxA*, and *GataE are known*, and *Smip *is a new gene that is expressed in the endoderm. The expression is strongly expanded in lithium-treated embryos (columns 3 and 4), whereas only at the most animal pole are ectodermal tissues left in the embryo. Blastula stage zinc-treated embryos do not exhibit any expression of endodermal markers (column 5). Gastrula stage zinc-treated embryos (column 6) do occasionally begin to express early endomesodermal markers as they recover from treatment (see Materials and methods). *Hex *is a transcription factor that is expressed at low levels in primary mesenchyme cells (PMCs) and predominantly in secondary mesenchyme cell (SMC) cells. Expression is upregulated in lithium-treated embryos, as determined by quantitative real-time polymerase chain reaction (Q-PCR; columns 3 and 4; compare with Table 1) but seems unchanged as determined by WISH and is eliminated in blastula stage zinc-treated embryos. P19 is a PMC-specific gene identified in the screen. Although its expression appears to be quantitatively upregulated in lithium-treated and zinc-treated embryos (see Table 2), WISH analysis indicates that the number of PMC cells forming is normal in lithium-treated or zinc-treated embryos, but that the PMCs migrate to the animal pole in lithium-treated embryos and to the vegetal pole in zinc-treated embryos. In neither case does a skeleton form.

Among the genes analyzed by WISH are many genes expressed in the endomesodermal domain, which have not yet been described (Additional data file 1). Among these are several transcription factors (genes encoding enzymes and suchlike are not described in detail here, but can be found in the WISH database [22]), including the following: *sox4*, *six3 *(Figure 3), *dlx *(Additional data file 1) and *six1 *(Figure 4), an ortholog of the *Hex *transcription factor family (Figure 5), *Lox, Dp-Hbn *(WISH database [22]), *Prox*, *Tbx6*, *snail*, and a *sox17 *ortholog (Figure 4). The *sox4 *and *six3 *genes have dynamic and opposing patterns of expression (Figure 3). Although *six3 *is expressed initially in the blastula stage at the animal pole, during gastrulation its expression is also restricted to the vegetal plate, forming a ring of expression around both poles of the early embryo. The *sox4 *gene, on the other hand, is expressed in the early blastula in the vegetal plate and is activated during gastrulation at the animal pole as well (Figure 3). *Tbx6 *is exclusively expressed in SMCs (Figure 4). Other interesting genes expressed in the vegetal components are a Smad-interacting protein and the c-*fos *transcription factor (Additional data file 1), which in vertebrates is a Wnt target gene and interacts with Smads [29].

**Figure 6:**
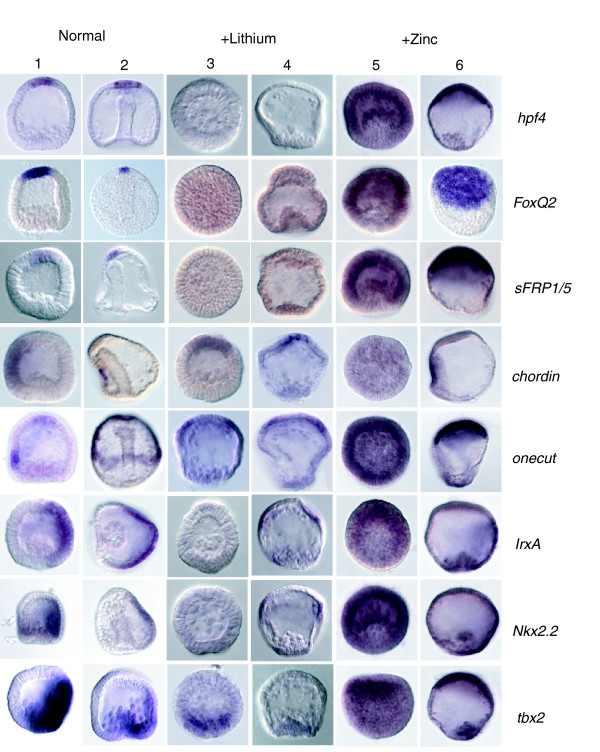
Expression of ectoderm markers in normal, lithium-treated, and zinc-treated embryos. Shown are whole-mount *in situ *hybridizations (WISHs) of ectodermal marker genes on blastula stage (columns 1, 3, and 5) and gastrula stage (columns 2, 4, and 6) sea urchin embryos. The genes under considerations are indicated on the right hand side. Expression of apical plate marker genes (*hpf4*, *FoxQ2*, and secreted frizzled protein 1/5 [*sFRP1/5*]) is lost in lithium-treated embryos (columns 3 and 4) and expanded in zinc-treated embryos (columns 5 and 6). Expression of the oral ectoderm marker *chordin *is shifted to the 'new' animal pole region in lithium-treated embryos (columns 3 and 4) but lost in blastula stage zinc-treated embryos (column 5). However, ectodermal differentiation does appear to take place in zinc-treated embryos if they are left to recover for a longer period of time (column 6). The ciliated band marker gene *onecut *exhibits wild-type-like expression in lithium-treated embryos, with a ring of expression around the animal pole (columns 3 and 4). The apical expression domain of *onecut *co-expands like the other apical organ markers in zinc-treated embryos (panels 5 and 6). Strikingly, the expression of aboral ectoderm markers (*IrxA*, *Nkx2.2*, and *tbx2*) is lost in blastula stage lithium-treated embryos (panel 3), whereas it is enhanced in zinc-treated blastula stage embryos, in which the expression appears to be quite uniformly distributed. *Tbx2 *is expressed in mesodermal cells and in the aboral ectoderm in normal embryos (columns 1 and 2). Strikingly, the ectodermal expression only is lost in lithium-treated embryos, whereas the mesodermal domain remains (compare with Figure 4).

Concerning the effect of lithium on the ectoderm, three observations were made. First, apical pole genes, which are those that are expressed at the animal most ectodermal region (such as *Fz5/8 *[15] and *SpNK2.1 *[30]), are eliminated. As shown in detail in Table 2, the expression ratios in lithium-treated embryos for newly discovered apical plate markers such as *FoxQ2*, *Zfhpf4 *(Figure 6), and *Dp-Hbn *(WISH database [22]) are 0.15, 0.01, and 0.05, respectively, which correspond to 6-fold, 100-fold, and 20-fold downregulation, respectively (as determined by Q-PCR). Second, the expression of oral genes is shifted to the animal side of the embryo, as was observed for *antivin*/*lefty *by Duboc and coworkers [16]. Third, genes expressed on the aboral side are strongly downregulated (Table 1). This is the case for the known transcription factor *tbx2 *(ratio 0.12, equivalent to a 8.3-fold downregulation) but also for the newly discovered aboral ectoderm transcriptional regulators *IrxA *(ratio 0.08, 12.5-fold downregulation) and *SpNkx2.2 *(ratio 0.15, 6.6-fold downregulation; Figure 6). Genes expressed in the oral ectoderm (*BMP2/4*, *lefty/antivin*, *nodal*, and *chordin*) or cilliary band (*Sponecut *and *SpPaxB*) are not clearly differentially regulated in lithium-treated embryos (Table 2; for insitus, see WISH database [22]).

Thus far, of a total of 700 genes that were analyzed by WISH, selected from either of the expression profiling experiments, 151 localized to an endomesodermal domain. We identified 34 clones restricted to primary mesenchyme cells (PMCs), 92 to SMCs, and 98 to ectodermal cells, of which about half co-localize to more than one cell type. About 400 genes exhibited ubiquitous expression or expression was too low to allow any detection. More than 2,400 images from these WISHs have been annotated, with the results accessible in the sea urchin WISH database [22].

### Zinc treatment expands the neuronal apical plate by downregulating vegetal signaling and oral markers, and upregulating aboral markers

**Figure 7:**
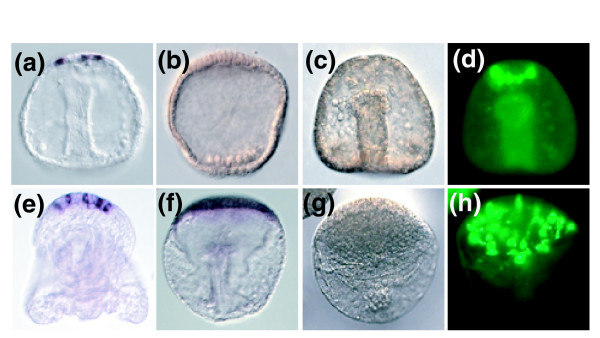
WISH analysis of *Glass *and *Mox *and immunohistochemical localization of serotonergic cells in normal and zinc-treated embryos. Whole-mount *in situ *hybridization (WISH) analysis identified the transcription factors **(a) ***Glass *and **(b) ***Mox *as being expressed in single cells of the apical organ. Although *Glass *expression is eliminated in zinc-treated embryos **(b)**, the expression of *Mox *is expanded and upregulated in zinc-treated embryos **(f) **(also see quantitative real-time polymerase chain reaction data in Table 2). Immunohistochemical localization of serotonin in **(c, d) **normal and **(g, h) **zinc-treated embryos shows that whereas normal embryos produce four to six serotonergic cells (panel d), the number of serotonergic cells is elevated to at least 30 on average in zinc treated embryos (panel h). Panels d and h are fluorescent photographs of the same embryos depicted in transmitted light in panels c and g, respectively.

**Figure 8:**
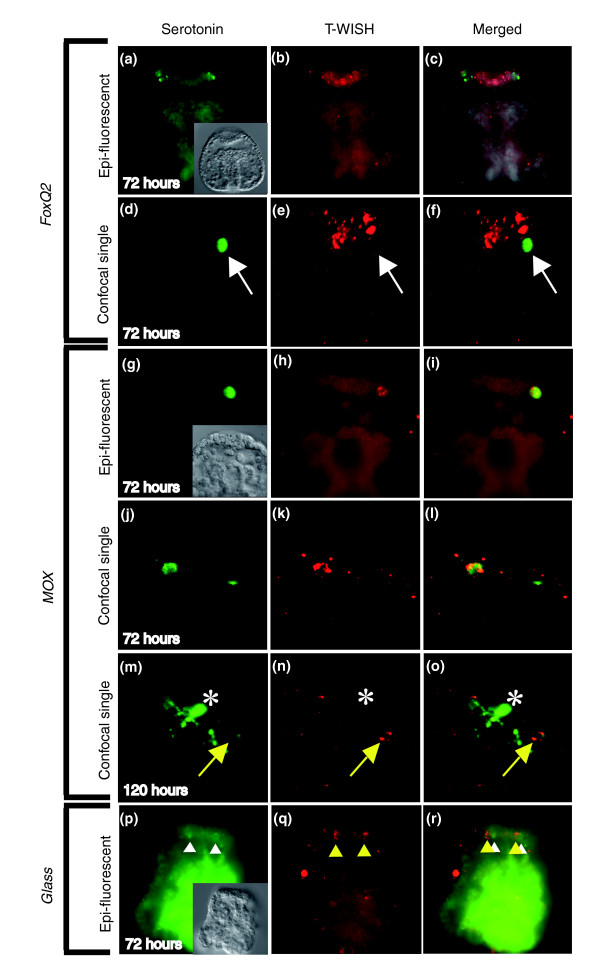
*Glass*, *Mox*, and *FoxQ2 *co-staining with serotonin. *FoxQ*2 mRNA is detected throughout the thickened neurogenic ectoderm at the animal pole of prisms (72 hours), but in 96 and 120 hour plutei there was no hybridization detectable. Tyramide amplification produces small foci of fluorescence in the cytoplasm of the cells that hybridize probe. There is diffuse background fluorescence throughout the remainder of the embryo. **(a to c) **In 72 hour prisms that have strong hybridization of the *FoxQ2 *probe to the neurogenic ectoderm, the anti-serotonin immunoreactive cells were localized outside the *FoxQ2 *region. **(d to f) **Single confocal optical section clearly shows serotonergic cells are *FoxQ2 *negative (white arrow). *Mox *mRNA was detected in the neurogenic ectoderm of prism and pluteus larvae. **(g to l) **In prism larvae, all of the serotonergic neurons were *Mox *positive. There are also some cells that are not immunoreactive with anti-serotonin, and they hybridize the *Mox *probe (not shown). **(m to o) **In plutei, neurons that are weakly immunoreactive with anti-serotonin hybridize with the *Mox *probe (yellow arrow). However, *Mox *mRNA was not detected in the neurons that strongly expressed serotonin. As the serotonergic neurons continue to differentiate during these stages, this may indicate that *Mox *is only expressed early in neurogenesis (asterisks in panels m to o). These preparations have relatively high background. **(p to r) ***Glass *mRNA appears not to co-localize with anti-serotonin immunoreactive cells in 72 hours prisms (white and yellow arrowheads).

The global view that arises from the analysis of this screen is that a majority of genes are downregulated in zinc-treated embryos. Zinc sulfate treatment has the opposite effect of lithium chloride and animalizes the embryos. No endomesoderm is formed and the embryos are 'arrested' as a hollow ball of ectodermal cells (Figures 1 and 5, 6, 7, 8). Zinc treatment is believed to have a nonspecific, purely inhibitory mode of action, which is in accordance with our findings. Nevertheless, there are two groups of genes that we found to be up-regulated. These are genes expressed in the apical plate and genes expressed in the aboral ectoderm. Table 1 shows that a majority of genes expressed in the vegetal plate are severely reduced in expression, indicating that vegetal signaling is largely blocked. Q-PCR analysis of Wnt genes indicates that all Wnts except *wnt1 *are expressed at significant levels (> 100 copies/embryo) at 38 hours (midgastrula stage; see Tables 1 and 3 and Figure 2). Of the ones that have significant (> 2-fold) differential expression, *wntA*, *wnt3*, *wnt6*, *wnt7*, and *wnt9 *are downregulated in zinc-treated embryos. Only one Wnt, namely *wnt8*, is upregulated in zinc-treated embryos. In addition, the secreted Wnt antagonist *sFRP1*/*5 *is markedly upregulated in zinc-treated embryos (Figure 6).

We found 14 genes that specifically localized to the animal plate, some of which appear to localize specifically to neuronal cells of the apical organ. The *Dp-Hbn *(WISH database [22]) gene is initially expressed broadly in the animal plate and becomes cleared during gastrulation from the central region, forming a ring of expression around the apical organ. A similar ring-like expression, embracing the developing apical organ, is also observed for the *six3 *gene, which is later also expressed on the vegetal side (see above). Several genes are expressed exactly in the apical organ. These are the transcription factors *FoxJ *(WISH database [22]),*FoxQ2 *(Figure 6), *Mox*, *glass *(Figure 7), a zinc finger gene (*hpf4*; Figure 6), a radial spoke protein, the tubulin β-chain gene (WISH database [22]), several genes without clear homology to any known genes, and - strikingly - *Sp-sFRP1/5*, which is a secreted frizzled protein (Figure 6).

We have analyzed three transcription factors (*FoxQ2*, *Mox*, and *glass*) for co-expression with serotonin and show here that the transcription factor *Mox *is specific for serotonergic neurons, whereas the transcription factor *glass*, which in *Drosophila *is required for the differentiation and survival of photoreceptor sells [31], localizes to cells adjacent to serotonergic cells (Figure 8). *FoxQ2 *and *Glass *are expressed in the neurogenic ectoderm but not in serotonergic neurons. Using the *FoxQ2 *gene as marker of the apical organ and *Mox *as a marker for serotonergic cells in zinc-treated embryos, we found that the few cells forming the apical organ in the sea urchin embryo are markedly expanded in the zinc-treated embryos (Figure 6), whereas this recently described new territory [30] appears to be entirely eliminated in lithium-treated embryos (Figure 6). Furthermore, we find that the expanded apical plate is extremely enriched in serotonergic neurons, where about 30 serotonergic neurons form, as opposed to five or six in normal embryos (Figure 7).

In addition to upregulation of genes of the animal plate or the apical organ, we also find a significant number of upregulated genes that are expressed in the aboral ectoderm in normal embryos. In fact, no transcription factor has yet been identified that is exclusively expressed in the aboral ectoderm. However, the fact that there is a cytoskeletal gene (*Spec2A *[32]) that is exclusively expressed in the aboral ectoderm does argue that such factors should exist (although post-transcriptional or combinatorial mechanisms of control of gene activity cannot be ruled out). As a control, we measured *Spec2a *expression in zinc-treated embryos and find that it is about tenfold upregulated (Table 2). One transcription factor that is expressed in the aboral ectoderm but that is also expressed in other territories is the T-box gene *Tbx2*/*3 *[15,33]. This gene was found to be significantly downregulated (Table 2; namely, clone RUDIREA_28I11, which is downregulated by a factor of 0.20; *P *= 3.50^e-12^) in lithium-treated embryos and is upregulated in zinc-treated embryos. We found two other transcription factors, namely *IrxA *(*Irx4/5*) of the Iroquois gene family and *Nkx2.2 *in the highly significant group in zinc-treated embryos. Both genes (as illustrated in Figure 6) are expressed in the aboral ectoderm, starting at very early stages, and expand their expression toward the oral side of the vegetal half during gastrulation in normal embryos. Hence, we propose that these transcription factors are essential components of the regulatory network that controls oral-aboral ectoderm differentiation. Because many aboral genes are upregulated in zinc-treated embryos, one would expect a downregulation of oral specific genes. This was found to be the case for the oral specific genes *chordin *(its antagonist *Bmp2/4*, also orally expressed, is not significantly differentially expressed) and *goosecoid*, but not for *nodal *and its antagonist *lefty *(see Discussion and conclusions, below).

## Discussion

Via a series of targeted array screens, we identified 250 genes exhibiting a restricted expression pattern. An analysis of global gene expression using whole-genome tiling arrays indicates that 9,000 genes are expressed in the sea urchin embryo [34]. Previous random WISH screens across multiple organisms have concluded that 20% of all genes assessed had a restricted expression pattern [35,36]. This could mean that perhaps 1,800 sea urchin genes are expressed in specific tissues during embryonic development. We hence assume that the genes identified thus far and the additional differentially expressed genes that have not yet been analyzed represent a significant portion of all tissue-specific sea urchin genes. This assumption provides the rationale for using our approach of combined array-WISH screens to unravel new candidate genes of GRNs, ultimately to move toward a global systems level understanding of sea urchin embryogenesis.

### Neuronal identity, apical plate, and zinc treatment

Among the genes that we found to be upregulated in zinc-treated embryos is the homeobox transcription factor gene *mox*, which is a member of the extended *hox *complex in humans [37], which in vertebrates has been found to be involved in mesoderm development [38,39]. By simultaneous WISH and immunohistochemical localization with serotonin, we could show that *Mox *is expressed in serotonergic neurons in the apical plate (Figure 8). Hence, this is the first transcription factor identified in sea urchin embryos that is expressed specifically by serotonergic cells; furthermore, its pattern of expression is consistent with its functioning in neuronal specification. It is also the first time that a *mox *ortholog had been found to be expressed by neurons in any organism. WISH analysis of *mox *in zinc-treated embryos revealed an apparent expansion of expression of *mox *in these embryos. Consistent with this, immunohistochemical localization of serotonin in zinc-treated embryos revealed an increase in the number of serotonergic neurons (Figure 7). Although two other transcription factors, expressed in the apical plate (*FoxQ2 *and *glass*), were found to be negative for expression in serotonergic neurons, it remains possible that they are expressed by one of the other types of neurons of the apical organ. The transcription factor *glass *is required for the differentiation and survival of photoreceptor cells in *Drosophila *[31]. In the sea urchin, *glass *is expressed in cells adjacent to serotonergic neurons. The structure of photoreceptors in sea urchins is not known, but it is presumed to involve sensory neurons and lack image-forming specializations. Thus, the apical organ may contain photoreceptors. However, there are no published data demonstrating that urchin embryos and larvae are responsive to photic cues.

The secreted frizzled-related protein gene *Sp-sFRP1*/*5*, selected because of being upregulated in zinc-treated embryos and downregulated in lithium-treated embryos, is also expressed exclusively in the apical plate and later in the apical organ (Figure 6). Secreted frizzled proteins are potent and highly specific inhibitors of Wnt signaling because they lack membrane domains and strongly compete with the Wnts on their receptors (frizzleds) [40]. This finding is an indication that downregulation of Wnt signaling may be a requirement for apical organ formation and neurogenesis, and one of the possible actions of zinc treatment on embryogenesis. A second finding, namely that aboral genes are upregulated in zinc-treated embryos, suggests that oral specific genes may be downregulated. This was found to be the case for the oral-specific genes *chordin *and *goosecoid*. However, other oral expressed genes exhibit a different pattern of regulation. As an example, the *chordin *antagonist *Bmp2*/*4 *is not differentially expressed, whereas *nodal *and its antagonist *lefty *are upregulated (see Q-PCR data in Table 2). This finding appears to contradict a recent finding that Nodal signaling, in the absence of vegetal signaling, represses the serotonergic cell content in the embryo [41]; hence, further investigation into the roles of BMP and nodal signaling, and expansion of the animal plate is required. The screens for zinc-treated embryos were conducted at a stage where normal embryos are at the gastrula stage (38 hours) and oral expression of *nodal *and *lefty *are downregulated in the oral ectoderm at this stage (expression shifts to the right side [42]). Hence, *nodal *and *lefty *may not be useful oral markers at this stage, and it is better to rely on *chordin *and *goosecoid*, which remain orally expressed until the end of gastrulation. Alternatively, there may be interactions between transforming growth factor-β signaling pathways, or the zinc-treated embryos may undergo a recovery process that leads to elevated expression of early patterning genes. Because many of the differentially expressed genes have not been analyzed in detail, we expect that there are additional genes that are involved in neurogenesis.

### TBX6, Notch, fibroblast growth factor, and Wnt signaling in SMC specification

In the sea urchin, induction of the mesodermal founder cells that give rise to the secondary mesenchyme cells of the embryo (SMCs; for example, pigment cells and blastocoelar cells) require a signal transduced by the Notch receptor [43-46]. This signal is the Delta ligand, which is expressed by the eight large micromere daughter cells beginning at the seventh cleavage [47]. The Delta signal is received directly by the adjacent cells of the macromere lineage. Our screen has identified fibroblast growth factor (FGF) signaling components *Tbx6 *and *snail *as components of SMC-specific gene expression (Figure 4). This signaling cascade reveals striking similarities in gene expression between sea urchin SMC cells and mouse presomitic mesoderm. In the mouse embryo *Tbx6 *is expressed in presomitic mesoderm during mouse gastrulation [48,49]. Studies have shown that Wnt signaling, in synergy with *T/TBX6*, controls Notch signaling by regulating *Delta1 *(*Dll1*) expression in the presomitic mesoderm of mouse embryos by demonstrating the need for T-box-binding and LEF/TCF-binding sites for activity of the *Dll1 *promoter in the tailbud and presomitic mesoderm. This suggests that *T/TBX6 *and Wnt signaling directly and synergistically regulate *Dll1 *transcription in the tailbud and presomitic mesoderm in mouse [50]. In addition, T-box transcription factors, as well as FGF and Wnt signaling, are essential regulators of formation, differentiation, and maintenance of paraxial mesoderm in mouse embryos, because mutations in T, *Fgfr1*, *wnt3a*, and *Tbx6 *cause defects in formation and differentiation of paraxial mesoderm [51-53].

Thus, it appears that several levels of crosstalk exist between the Notch, the Wnt, and the FGF pathways in somitogenesis in the mouse. The co-expression of *Tbx6*, *FGFR1*, and *FGFR3 *(this report) and *delta *[54] in sea urchin embryo suggests that during sea urchin SMC specification, differentiation, or maintainance, highly similar processes function as in mesenchymal epithelial transition of mouse presomitic mesoderm to somites. Hence, we propose that in sea urchin there may exist a feed-forward loop, in which *Tbx6 *and Wnt may act in synergy to activate *delta *to control Notch signaling in SMC differentiation. Moreover, we also found the transcription factor *Prox1 *to be co-expressed with *FGFR3*. This indicates that, like in mouse, *FGFR3 *may be a target of *Prox1 *[55]. Interestingly, a *PKCdelta1 *gene is expressed in migrating SMC cells. In the *Xenopus *embryo it has been shown that *PKCdelta *is essential for dishevelled function in a noncanonical Wnt pathway that regulates convergent extension movements [56]. This indicates that noncanonical Wnt signaling is involved in cell migration and convergent extension movements during sea urchin embryogenesis and further indicates that there exists crosstalk between Wnt, Notch, and FGF signalling in secondary mesenchyme (SMC) specification and differentiation.

### Evolution of axial patterning

In the development of bilaterian animals, the indirect mode of development is regarded to be ancestral because it occurs in deuterostomes (echinoderms and hemichordates) and protostomes (lophotrochozoans), but in chordates (and ecdysozoan protostomes) indirect modes of development appear to have been lost during evolution [57]. Indirect development (also referred to as maximal indirect development) is characterized by use of a larval stage, the body patterning of which is essentially different from the body patterning of the adult [57]. The comparative study of gene expression patterns in the larvae of protostomes (for instance, annelid trochophora) and deuterostomes (for example, sea urchin dipleurula type) is a key step toward gaining insights into the Urbilateria, the common bilaterian ancestors. For example, comparison of gene expression between three patterning genes in indirect developing lophotrochozoan embryos and their counterparts in vertebrates and basal deuterostomes suggests that the Urbilateria has developed through a free swimming larva [58].

Gene expression data in lophotrochozoan larvae are still few, and gene expression studies in indirect developing basal deuterostomes are also in their infancy; hence, this study aims to close the gap.

There are several parallels between the organizer region of other animals and the vegetal pole of sea urchin. For example, in all cases this is the site of gastrulation as well as the source of axial specification signals that are capable of reorganizing surrounding tissue upon transplantation. *Chordin *and *nodal *are molecules classically associated with organizer and dorsal axis specification; in particular, *chordin *was used to interpret the expression on the ventral side in flies as possible proof of an axis inversion that happened during the evolution of tribloblastic animals. In fact, ecdysozoans are very derived animals with a direct mode of development that is regarded not to be ancestral [57]. The derived state of ecdysozoans is reflected by the recent finding that diploblastic cnidarians may share more genes with vertebrates than they do with ecdysozoans [59]. In sea urchin embryo we have an example in which the gene expression pattern of *chordin *and the organizer-like region are dissociated. One could view this finding as an indication that the vegetal plate is not the only organizer region in sea urchin embryo, but that the oral ectoderm also has organizer functions, perhaps similar to head and trunk organizer in vertebrates. Moreover, the classical dorso-ventral patterning genes *chordin *(Figure 6) and *nodal*, and their antagonists *BMP2*/*4 *and *lefty*/*antivin *[16,42], respectively, are all expressed at the same time on the same side of the embryo, in the oral ectoderm, and this may have further implications for the evolution of axial patterning. Even more strikingly, a recent analysis of dorso-ventral genes in the cnidarian *Nematostella vectensis *has revealed a similar situation in which, unlike in flies and vertebrates, the transforming growth factor-β ligands and their antagonists are co-localized at the onset of gastrulation in presumptive endoderm [60].

It is therefore imperative to analyze the development of indirect developing organisms in order to analyze axis relationships between protostomes and deuterostomes.

We predict that *chordin *will be expressed on the ventral side of lophotrochozoan embryos as well, which would indicate that the bilaterian ancestor developed through a swimming larva, similar to types of larvae that can be found in basal deuterostomes, such as echinoderms and hemichordates and prototypic protostomes, the lophotrochozoans.

Such a result would also mean that an axis inversion could not have taken place at the time point in evolution when the tribloblastic world arose, but rather at the time when chordates arose. Expression and GRN architecture analysis of the *chordin *molecule and other dorso-ventral patterning genes in lophotrochozoan embryos and in amphioxus will clarify this interesting aspect of bilaterian evolution.

## Materials and methods

### Embryo cultures and treatments

The fertilized eggs were grown at a maximal density of 1 to 2 × 10^7 ^eggs/liter (1% to 2% volume; 1 ml settled eggs roughly equals 10^6 ^eggs/embryos, so no more than 10,000/ml) in filtered seawater in 1 to 3 liter beakers. The antibiotics penicillin (20 units/ml) and streptomycin (50 μg/ml) were added to cultures that were to be grown for longer than 15 hours. The cultures were kept at 16°C with continuous stirring.

For lithium chloride treatment embryos were cultured in seawater containing a 30 mmol/l end concentration of lithium chloride, which was added after egg activation (namely at the two-cell stage). For expression profiling we used the midblastula stage at 20 hours of development. Zinc sulfate treatments to animalize the embryos were performed as described by Nemer and coworkers [61]. Expression profiling was performed on 38-hour embryos.

### WISH, TWISH, and immunohistochemical localization of serotonin

Fixation of embryos and WISH was performed in accordance with the method described by Minokawa and coworkers [62] or as described previously [63]. Tyramide signal amplification (TSA) with whole mount *in situ *RNA hybridization (TWISH) and immunohistochemical localization of serotonin was performed in accordance with the method described by Yaguchi and Katow [64]. The anti-serotonin antibody used was made in rabbits and obtained from Sigma (Munich, Bavaria, Germany) (product number S5545). As secondary antibodies we used anti-Rabbit (IgG)-Alexa594 (red) from Molecular Probes (product number A-11037) and anti-Rabbit (IgG)-Alexa488 (green) from Molecular Probes (Eugene, OR, USA, product number A-11034).

### Expression profiling

Plasmid inserts of 35,238 cDNA clones representing about 20,000 genes of the sea urchin *Strongylocentrotus purpuratus *[21] were PCR amplified in a 30 μl volume in 384-well plates. PCR was performed in 1 × PCR buffer, with 0.1 mmol/l primer, 200 mmol/l of each dNTP, 1.5 mol/l betain, and 2 to 3 units Taq polymerase. As vector specific primers, M13 forward: 5'-GCTATTATGCCAGCTGGCGAAAGGGGGATGTG-3' and 3/86: 5'-CCGGTCCGGAATTCCCGGGT-3' were used to amplify the inserts. Ready PCR mix was inoculated with a bacterial suspension using a replicator. PCR was performed for 30 cycles with the following cycle profile: 20 s denaturation at 94°C followed by a one-step 210 s annealing and extension step at 65°C.

DNA was transferred to dry Hybond N+ Nylon membranes using in-house build spotting robots. Filters were fixed to tiles using sticky bands. Each DNA was spotted seven times. Pin diameter used was 250 μm, and the spot distance was 900 μm in the common 5 × 5 pattern (each clone in duplicate) [65]. After spotting filters were denatured by 2 × 5 min soaking on whatman paper weatend with 0.4 mol/l NaOH followed by 2 × 5 min denaturation on whatman paper soaked with 0.5 mol/l NaH_2_PO_4 _(pH 7.2). Filters were air dried overnight and cross-linked twice at 12 J/cm^2 ^using the UV Stratalinker 2400 (Stratagene, La Jolla, CA, USA).

Embryos were dissociated in 20 volumes Trizol (Gibco BRL Life Technologies, Rockville, MD, USA), shockfrozen in liquid nitrogen, and stored at -80°C until use. For RNA isolation the sample was defrosted in a 65°C waterbath, 0.2 ml chloroform/ml Trizol was added, and the mixture was shaken for 15 s, followed by an additional 5 min at room temperature. The samples were subsequently balanced and then centrifuged at 12,000 *g *for 15 min at 4°C in a Sorvall SS34 rotor in silinized RNAse free glass tubes. Supernatant was transferred to a new RNAse free tube and 0.5 ml isopropanol/1 ml Trizol added. After thorough mixing, the sample was stored for 10 min at room temperature followed by a 10 min spin, as above. The RNA pellet was then washed with 20 ml of 75% ice-cold ethanol and spun for 5 min at 7,500 *g *at 4°C. The pellet was air dried for 10 min and re-suspended in 500 μl RNAse free water. The integrity of the RNA was analyzed by gel electrophoresis and its concentration was determined spectrophotometrically. RNA was aliquoted and stored at -80°C.

For poly A selection, the PolyATract mRNA Isolation System III (Promega, Mannheim, Germany) was used, in accordance with the manufacturer's instructions. Usually, 8 to 10 μg poly A RNA was obtained from 1 mg total RNA. RNA concentration was determined spectrophotometrically. A typical labeling reaction was carried out with 1 μg poly A RNA (used for two filters). One microgram of poly A RNA concentrated in a volume of 8 μl was mixed with 1 μl of random primer hexamers (Gibco BRL; 2.5 μg/μl), denatured for 10 min at 70°C, and immediately placed on ice. Subsequently, the following was added: 1 μl RNasin (Promega), 1.5 μl cMix, 6 μl 5 × reverse transcriptase buffer, 3 μl 0.1 mol/l DTT, 7 μl α- [P-^33^] dCTP (70 μCi), and 2 μl Superscript II reverse transcriptase (Gibco BRL). The mixture was mixed well and incubated at 37°C for 2 hours. Incorporation was measured by running 1 μl on a PEI paper followed by capturing intensities by scanning on a phosphorimager. ImageQuant software (GE Healthcare, Munich, Germany) was used to measure the ratio of incorporated versus unincorporated nucleotides. The rate of incorporation is usually more than 90%. Probe was denatured by addition of 10 μl 5 mol/l NaOH. Hybridizations were carried out in modified Church buffer at a probe concentration of 100 ng/ml in a 10 ml volume, with two filters separated by a nylon mesh in one bottle at 65°C for 20 hours. Bottles were carefully cleaned with 1 mol/l NaOH before use. After hybridization, filters were washed four times in 1 l wash solution containing 40 ml of 0.5 mol/l NaH_2_PO_4 _(pH 7.2), 10 ml 10% SDS, and 2 ml 0.5 mol/l EDTA, twice at room temperature and twice at 65°C. Filters were then wrapped in saran wrap without any wrinkles, exposed for 10 hours, and scanned at 100 μm resolution using the Fuji BAS 1800 phosphorimager.

### Statistical analysis

Gene expression data were normalized as described previously [66]. The validity of gene expression of each individual signal was judged by comparison with a negative control sample. In order to verify whether a given gene was significantly expressed, we compared its signal with a signal distribution derived from negative controls. In our array design, we distributed about 6,000 empty spot positions on the array. After quantification, a small, non-zero intensity was assigned to each empty spot, reflecting the amount of background signal on the array. Because these positions were spread uniformly over the array, the distribution of signals reflects a global background distribution for the experiment and indicates whether cDNA signals were at or above the background level of expression. For each cDNA, we counted the relative proportion of empty positions on the array that were smaller than the observed intensity (background tag [BG]). Background-tags from replicated experiments for the same cDNA were averaged. Thus, high values (close to 1) indicated that the cDNA was expressed in the tissue tested, whereas low values reflected noise. cDNAs were considered 'expressed' when their average background-tag was above 0.9, a threshold consistent with the limit of visual detection of the spots.

For each cDNA, we performed statistical tests based on the replicate signals in experiments with treated and untreated samples. Four standard tests were used in parallel: Student's *t*-test, the Welch test, Wilcoxon's rank-sum test, and a permutation-based test [24].

To analyze pathways based on gene expression signals, we tested whether the entire group of genes associated with specific pathways exhibited differential expression across treated and untreated conditions. We mapped the sea urchin genes via the glean3 protein predictions of the sea urchin genome [25] to the human ensemble genes (National Center for Biotechnology Information version 36) and those to HUGO via reciprocal blast. Symbols that are grouped into Kyoto Kyoto Encyclopedia of Genes and Genomes pathways (version 18.05.2006) were used to analyze pathway-specific differential regulation of sea urchin genes. The procedure computes a nonparametric test for the groups of genes organized in pathways and judges which pathways are affected by the treatment. This is described in detail elsewhere [67]. All array data can be found in the Array Screens Database [23].

### Quantitative real-time PCR

Total RNA was isolated from embryos subjected to 20 hours of lithium treatment embryos and from those subjected to 38 hours of zinc treatment, as well as from untreated embryos at corresponding time points by extraction with Trizol (Gibco BRL), following the manufacturer's instructions. The integrity of the resulting RNAs was analyzed by gel electrophoresis. In case of DNA contamination, RNAs were DNAse treated using the TURBO DNA-free^® ^Kit (Ambion, Austin, TX, USA). cDNA was transcribed with random hexamers and 1 μg total RNA using M-MLV reverse transcriptase (Promega). The cDNAs were used as Q-PCR templates to determine mRNA transcript levels of several genes in embryos at the embryonic stages mentioned above. Quantitative real-time PCR measurements were performed on an ABI 7900 HT Detection System using SYBR Green PCR Master Mix (ABI, Foster City, CA, USA) with the following thermal cycling parameters: 50°C for 2 min and 95°C for 10 min, followed by 40 cycles of 95°C for 15 s and 60°C for 1 min. To determine the expression of a specific gene, *SpZ12-1 *amplification for normalizing measurements of the absolute number of transcripts of the gene as well as *ubiquitin *amplifications as an amplification reliability standard were also carried out on the same sample. Each PCR reaction was performed in triplicate.

A threshold was arbitrarily set within the exponential range of the amplification process so that different samples could be compared in terms of the number of cycles required to attain the threshold (CT, threshold cycle) when considering the same marker gene. The Q-PCR primer sequences used to determine the amount of transcripts of a specific gene can be found in Additional data file 2. Gel electrophoresis and dissociation curve were used to confirm product. Primer efficiency was found to be around 1.96. The average CT value obtained for the gene of interest (CT_GOI_) was normalized to the average CT value acquired on the same control cDNA preparations with *SpZ12-1 *primers (CT_SpZ12_): ΔCT = CT_GOI _- CT_SpZ12_. The exact number of transcripts per untreated embryo of the specific gene at 20 hours and 38 hours respectively (Q_GOI_) was calculated using the known values described by Wang and coworkers [68] and Minokawa and colleagues [62] for *SpZ12-1 *at the same stages (Q_SpZ12_): Q_GOI _= Q_SpZ12 _× 1.96^-ΔCT^.

The ratio of gene expression between treated and untreated embryos was calculated as follows. First, the average CT value of the specific gene (CT_GOI_) was normalized to the average CT value acquired with ubiquitin primers (CT_Ubq_) on cDNA obtained from untreated embryos (ΔCT [con] = CT_GOI _[con] - CT_Ubq _[con]) as well as on cDNA obtained from treated embryos (ΔCT [tre] = CT_GOI _[tre] - CT_Ubq _[tre]). The ratio was then determined by using the difference in normalized CT values: r_GOI_(tre/con) = 1.96^ΔCT(con)-ΔCT(tre)^. To determine the exact number of transcripts of the specific gene per treated embryo, the ratio found was multiplied by the exact number of transcripts of the same gene in the control embryo, as calculated previously: Q_GOI_(tre) = r_GOI_(tre/con) × Q_GOI_(con). The numbers given in the figures and tables are averages of the results of at least two independent experiments using two different batches of cDNA.

### Databases and database management

Development and maintenance of the sea urchin web application was done on a dual core 64 bit computer running a Linux operating system using an Apache webserver [69]. The database was implemented with a relational sqlite3 database [70]. The web interface was created dynamically by CGI scripts [71] written in the Perl programming language [72], and Javascript components were used to enhance the user interface. Connection to the database is done via the DBI module [73] and the DBD::Sqlite module [74]. All data that accompany this report can be found in the sea urchin EST database [75], the Array Screens Database [23], the WISH database [22], and the genome database [76]. The sea urchin genome annotation data are accessible via the Baylor College Genome Project annotation database [25,77].

## Additional data files

The following additional data are available with the online version of this paper. Additional data file 1 provides a figure with WISH images of 88 endomesoderm genes in *Strongylocentrotus purpuratus *embryos at blastula stage (20 hours). Additional data file 2 provides a table summarizing the sequences of the used Q-PCR primers, amplicon size, and melting temperatures.

## Supplementary Material

Additional data file 1 Provided are WISH images of 88 endomesoderm genes in blastula stage *Strongylocentrotus purpuratus *embryos (20 hours). Click here for file

Additional data file 2 Provided is a table summarizing the sequences of the used Q-PCR primers, amplicon size, and melting temperatures. Click here for file
