# Supplementary material for: A global view of gene expression in lithium and zinc treated sea urchin embryos: new components of gene regulatory networks
Source: Genome Biol. 2007 May 16;8(5):R85. doi: 10.1186/gb-2007-8-5-r85 (PMC1929154; doi:10.1186/gb-2007-8-5-r85)
Supplement: Additional data file 1 — Provided are WISH images of 88 endomesoderm genes in blastula stage Strongylocentrotus purpuratus embryos (20 hours). [file gb-2007-8-5-r85-S1.pdf]

Fig. S1: WISH images of new endomesoderm genes in blastula stages

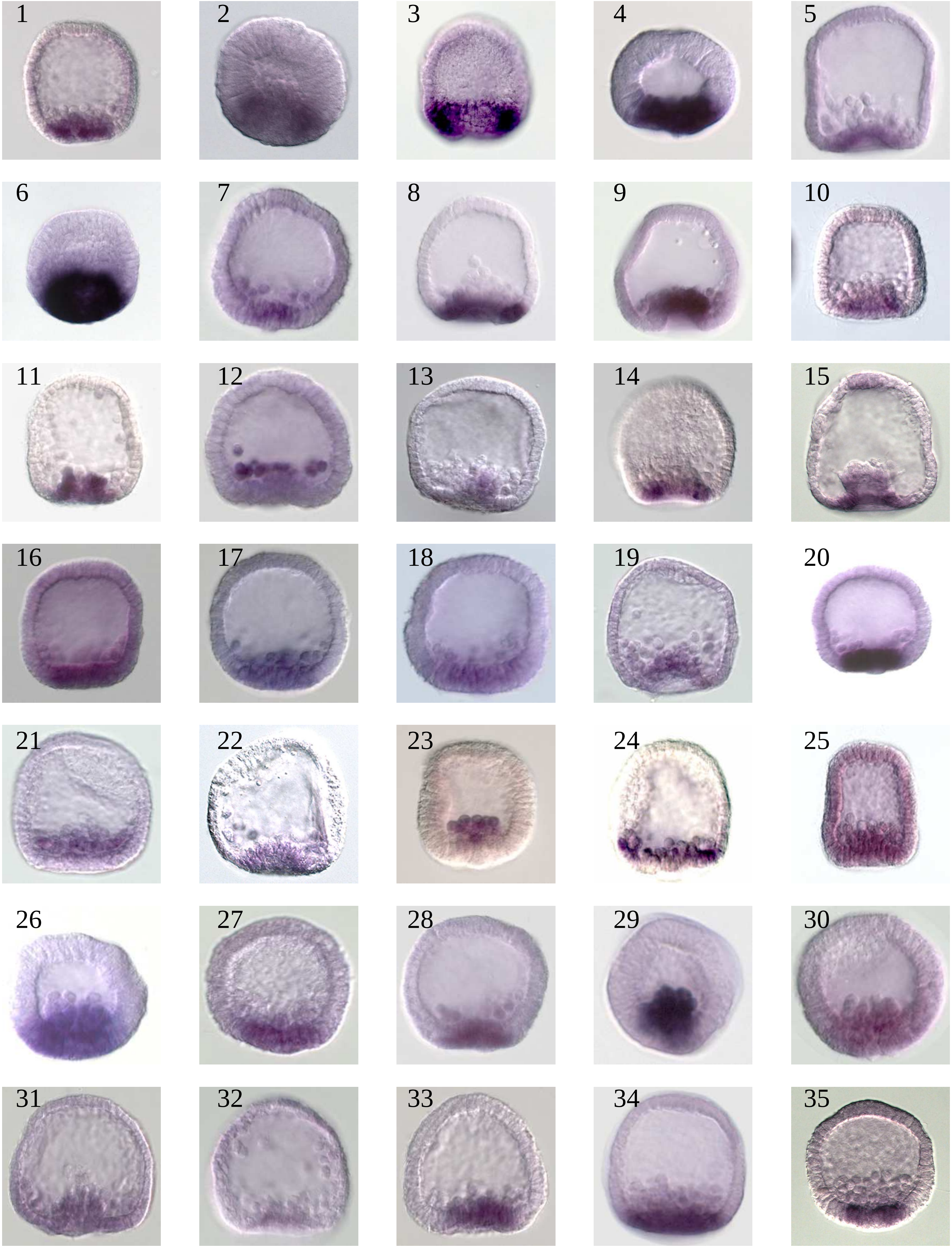

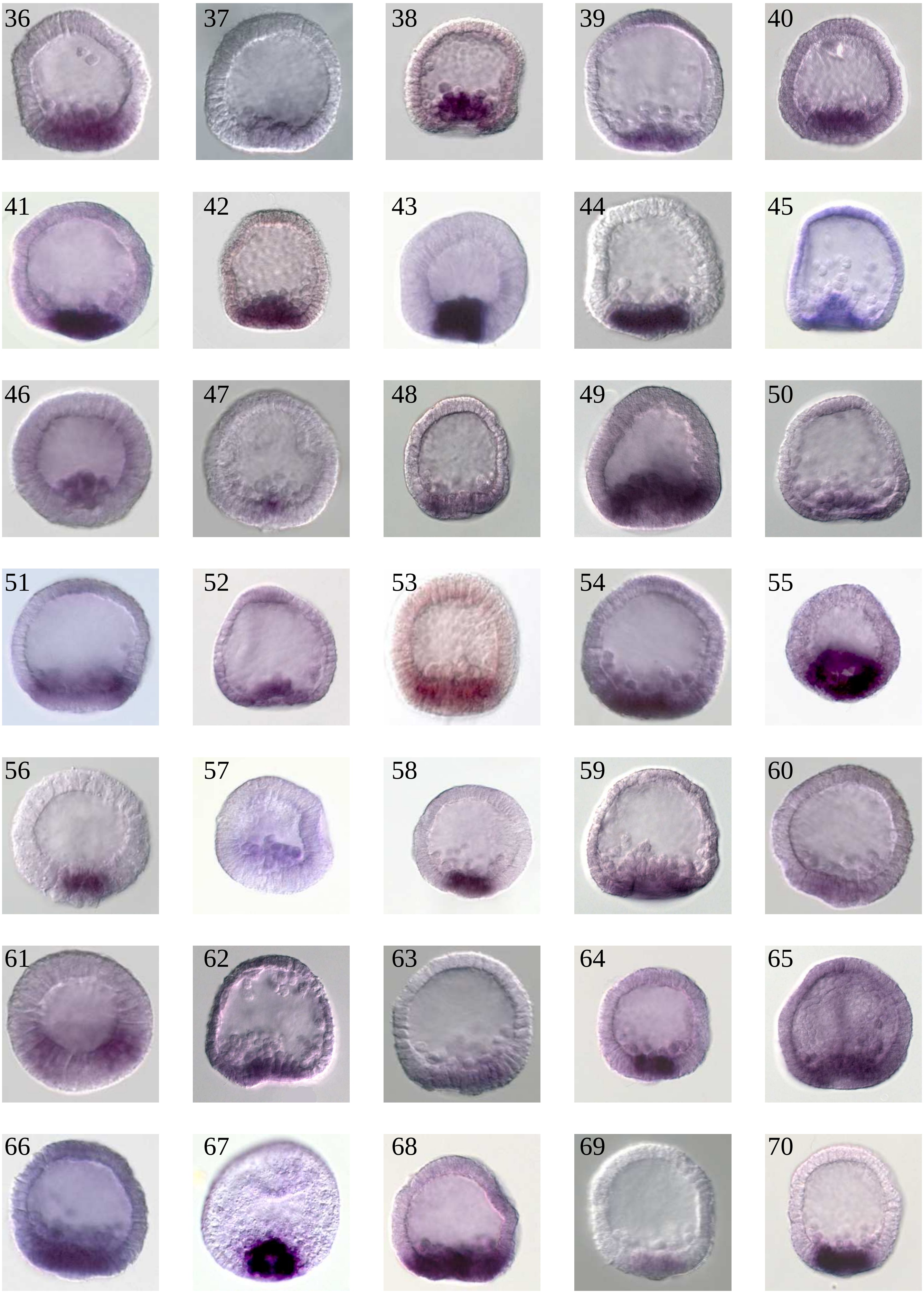
36
37
38
39
40
41
42
43
44
45
46
47
48
49
50
51
52
53
54
55
56
57
58
59
60
61
62
63
64
65
66
67
68
69
70

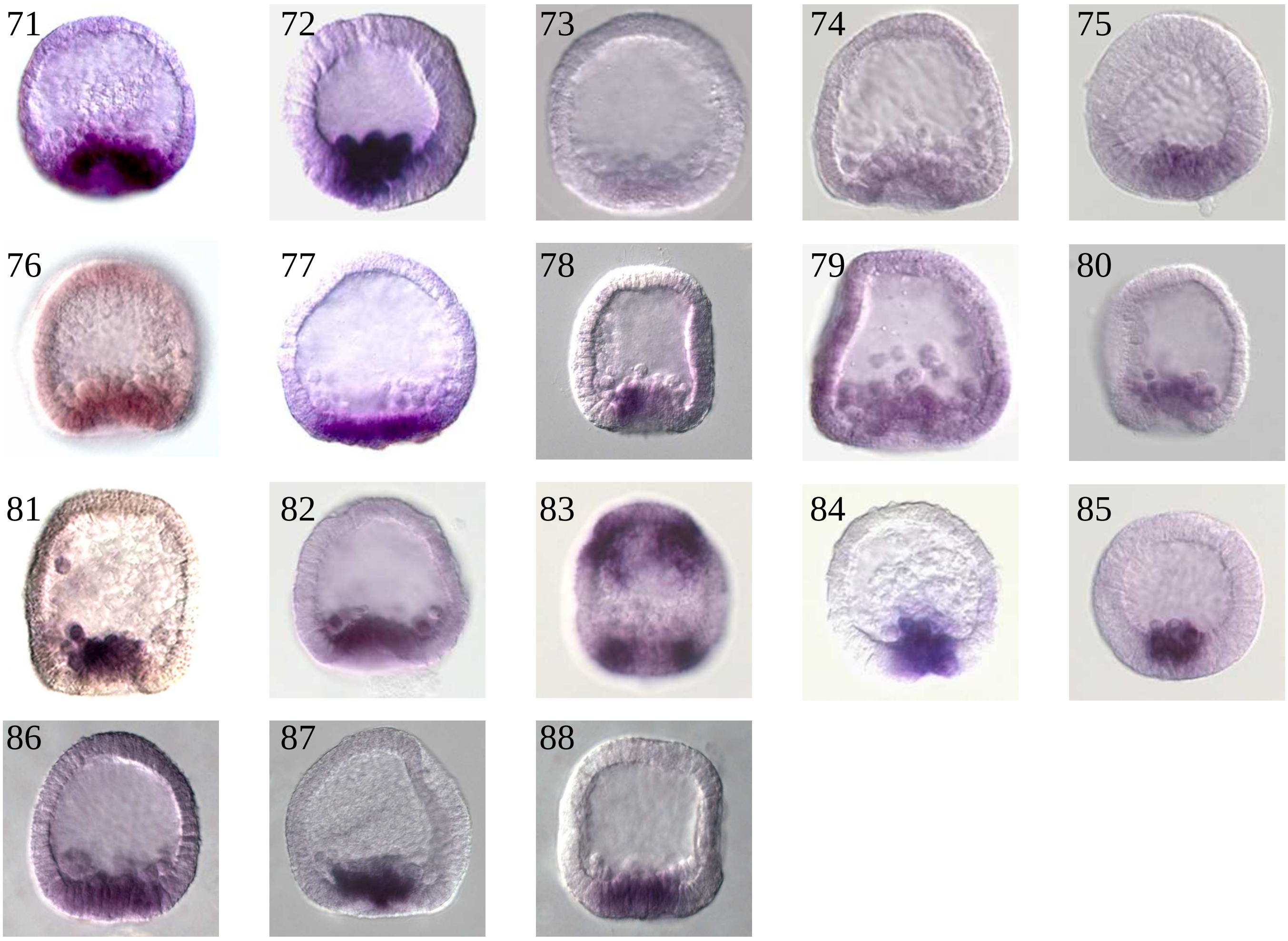

Clone names that can be used to query the WISH database and gene Ids are:
01 388_536REAsu4_4G6_blas2a.jpg similar to homeobox nanog? Or Adhesive plaque matrix protein VLPV
02 125_537REA_12I5_Blas_1a.jpg avidin
03 611_538REA_4O4_blas_1b.jpg dachshund
04 538_RUDIREA_2H10_14hr1d.jpg sox4
05 130_538REA_10H13_EGas_2a.jpg dead boix rna helicase
06 541_537REA_2L11_blas_2a.jpg putative polyprotein
07 417_RUDIREA_5P3_LBlas_1a.jpg
proline serine threonine phosphatase interacting protein 1
08 714_RUDIREA_33H6_LBlas_2b.jpg smad interacting protein
09 599_RUDIREA_36N15_EGas_2a.jpg Sp-HNRPR
10 339_536REAsu4_17M6_blas_1a.jpg hypothetical protein human flj30760
11 340_536REAsu4_13J21_Egas_3a.jpg unknown serine rich
12 257_RUDIREA_24K20_LBlas_2a.jpg microtubule associated protein
13 635_538REA_3N5_LBlas_2a.jpg protein-tyrosine phosphatase alpha precursor
14 357_RUDIREA_2B6_blas_1a.jpg paraneoplastic neuronal antigen ma3
15 230_Rudirea_20C4_EGas_1a.jpg Frzb1
16 706_621REA_1N13_LBlas_1a.jpg amnionless protein precursor
17 455_RUDIREA_33A16_LBlas_1a.jpg pol polyprotein
18 453_RUDIREA_21N17_LBlas_1a.jpg elongation factor 1 gamma
19 569_RUDIREA_41G14_EGas_1a.jpg Sp-Vdac2
20 575_536REAsu4_16C15_Blas_2a.jpg Sp-Nit1

21 557_RUDIREA_28A7_LBlas_2a.jpg talin
22 016_537REA_15E20_30hr_1aX.jpg meta1
23 368_536REAsu4_14E6_Mblas_2a-17.jpg Sp-Fmo3
24 454_RUDIREA_26N21_20h_1aX.jpg Sp-Carbonic anhydrase-4-like-B
25 159_621REA_7A10_24hr_3aX.jpg Ariadne RNA binding protein
26 546_537REA_7F17_Lblast_8a.jpg Sp-Dao1
27 508_538REA_9C21_LBlas_1a.jpg mili (miwi like)
28 684_537REA_12B16_Blas_2a.jpg cofilin
29 472_536REAsu2_6M17_Blas_1a.jpg Sp-cyclophilin
30 667_536REAsu4_14A22_LBlas_2a.jpg B-cell translocation gene
31 602_RUDIREA_31P4_EGas_1a.jpg signal sequence receptor beta subunit
32 679_RUDIREA_2D4_LBlas_1a.jpg
sparc precursor (secreted protein acidic and rich in cysteine)
33 517_RUDIREA_20I10_LBlas_2a.jpg Sp-Acads
34 474_536REAsu2_8F9_LBlas_1a.jpg Sp-Nova1
35 237_Rudirea_19K21_LBlas_2a.jpg fimbrin
36 101_536REAsu2_6H4_LBlas_1a.jpg Sp-AN-like4
37 644_RUDIREA_14F18_LBlas_1a.jpg Transcription factor NTF-1 grainy head
38 436_RUDIREA_18I10_blas2a.jpg Sp-Cathepsin2
39 696_RUDIREA_31D14_LBlas_3a.jpg 40s ribosomal protein s2
40 240_Rudirea_21o23_LBlas_1a.jpg Sp-Six1/2
41 632_RUDIREA_10M1_Lblas_1a.jpg Sp-Hadha , 78 kda gastrin-binding protein
42 338_536REAsu4_14H13_Lblas_1a.jpg Sp-TRIO
43 550_537REA_15K13_Eblas_1a.jpg Sp-P19
44 703_537REA_9G14_LBlas_3a.jpg DMBT1
45 529_537REA_6J13_Egast_1a.jpg Sp-seawi
46 253_RUDIREA_21G8_Blas_1a.jpg microtubule associated protein PMC
47 658_RUDIREA_2L21_LBlas_3a.jpg 70 kda heat shock protein
48 603_RUDIREA_33E9_Lblas_2a.jpg Sp-Dmgdh
49 146_Rudirea_33K5_LBlas_3a.jpg fibropellin-like
50 419_Rudirea_9E15_EGas_1a.jpg Sp-Slc7a11 Cystine/glutamate transporter
51 451_RUDIREA_1B4_LBlas_1a.jpg SpMLCK myosin light chain kinase
52 694_RUDIREA_9I20_EGas_1a.jpg repeat?
53 155_536REAsu2_4L15_21hr_1aX.jpg Sp-Hmgcr Coenzym A reductase
54 675_RUDIREA_29E16_Blas_2a.jpg 90-kda heat shock protein hsp83
55 649_RUDIREA_27A7_Blas_1b.jpg, Gypsy polyprotein
56 681_621REA_1B17_Blas_2a.jpg presynaptic protein sap97
57 537_RUDIREA_36F9_Lblast_1a.jpg
Sp-WAP/IG growth and differentiation factor-associated serum protein 1
ADHESION PROTEIN Sp-WAP/IG/KU/C345C
58 585_RUDIREA_25B3_blas_1a.jpg peroxisomal bifunctional enzyme Sp-Ehhadh
59 394_Rudirea_28G3_EGas_1a.jpg Sp-Slc7a11 Cystine/glutamate transporter
60 512_RUDIREA_39H17_LBlas_1a.jpg
eukaryotic translation initiation factor 3 p42 subunit Sp-EIF3S4
61 92_536REAsu4_17J2_Blas_1a.jpg dlx
62 725_621Rea_14C17_LBlas_2a.jpg SuH
63 689_538REA_10O19_LBlas_2a.jpg
Sp-eIF3S5-like eukaryotic translation initiation factor 3 subunit 5
64 221_RUDIREA_13L18_blas1a.jpg snail
65 381_537Rea_13B9_LBlas_2a.jpg transcription elongation regulator 1
66 118_536REAsu4_13G16_LBlas_1a.jpg Flavin containing monooxygenase 6
67 662_536REAsu4_1F23_Mgast_3a.jpg penicillin binding protein ?
68 175_537REA_13G10_blas1a.jpg
ubiquitin ligase protein mib2 putative nf-kappa-b-activating protein mind bomb2
69 677_537REA_6D22_Blas_1a.jpg Sp-Aldoaa fructose-bisphosphate aldolase 2
70 219_RUDIREA_15N17_Eblas3a.jpg Prox1

71 645_RUDIREA_17J13_Egast_2b.jpg pol-like peptide
72 260_RUDIREA_24K14_Egast_3a.jpg c-fos
73 688_537REA_16H4_LBlas_4a.jpg Sp-Cant1 apyrase

74 617_537REA_16I22_EGas_1a.jpg
eukaryotic translation initiation factor 3 subunit 6
75 570_RUDIREA_34J12_Blas_3a.jpg
Sp-carboxypeptidaseD-like carboxypeptidase d
76 144_RUDIREA_28K19_24hr_1aX.jpg Chaperonin precurser
77 651_RUDIREA_30O18_Lblast_4a.jpg
retinitis pigmentosa 2 ?? GLEAN3_26946 Scaffold90366
78 403_RUDIREA_29D01_LBlas_2a-28.jpg Tbx6
79 672_RUDIREA_39G20_LBlas_1a.jpg vacuolar sorting protein 35
80 423_RUDIREA_12G12_LBlas_1b.jpg Sp-ELAV-like elav-like3
81 062_RUDIREA_14B4_30hr_1a_VX.jpg polycystin-like2
82 600_RUDIREA_37F4_Blas_2a.jpg lysine hydroxylase
83 245_SpSMBLAS_47F18_Egas2b.jpg six3
84 297_SpSMBLAS_127O24_Lblast_1a.jpg Sp-papss
85 244_SpSMBLAS_124N22_Eblas1a.jpg Hex
86 461_536Reasu4_3B3_LBlas_1a.jpg Neuro-oncological ventral antigen 1
87 720_Rudirea_21I02_LBlas_3a.jpg Sp-astacin protease 1
88 460_536Reasu4_1D7_LBlas_3a.jpg alpha-mannosidase
