# Supplementary material for: A global view of gene expression in lithium and zinc treated sea urchin embryos: new components of gene regulatory networks
Source: Genome Biol. 2007 May 16;8(5):R85. doi: 10.1186/gb-2007-8-5-r85 (PMC1929154; doi:10.1186/gb-2007-8-5-r85)
Supplement: Additional data file 2 — Provided is a table summarizing the sequences of the used Q-PCR primers, amplicon size, and melting temperatures. [file gb-2007-8-5-r85-S2.pdf]

Table S1: Sequences of used Q-PCR primers

GENE	FORWARD SEQ	REVERSE SEQ	SIZE	PRODUCT Tm
β-Catenin	CAGAGCAATGCCACCTCACA	CCCAGTCAAACATGCCCTG	109 bp	85
ADP-Ribosylcyclase	TGCCTACATACCCCATGACGT	GCGCAGAAAAGGACAAGTTCA	101 bp	81
AEX3	ACGTCGCTTACCCCCACTACT	GTGATGGCTGGAGAGAGAGCA	106 bp	82
Alx	ACCCACCACTCCGTACCATG	CTCGGGCTATCTCTTCCCG	104 bp	80
Arginine kinase	AGATTGGGAACCTCCGAGGT	TCGATGGGCTCTCCTTTCTC	101 bp	79
Blimp1	GACGACGAAGGCAAGTTGATC	TTGAGACGTTTCGTCCATGG	102 bp	80
BMP2/4	CAACTGCAGACGCCATGAAT	CACCGCGACAATAGTAGGCC	101 bp	81
Brachury	TGGCAGTATCTCCCTCCTCG	CCACATGCTGCCGTATTGACT	103 bp	83
Chordin	CGGTGGTGATGTTGTCAAGGT	AGGTTCGGATGCCACTCATCT	150 bp	81
CoA-reductase	CTCAGTGTGAGGTGGTGGACA	GGTCCCGATGCTGATGACC	104 bp	84
Delta*	ACGGAGCTACATGCCTGAAC	TCACAATGGACCGAATCAGA		
DLX	ACCCAGAGGCTCCCAGTCA	GCAGTTGTGGTTGCTGAGGTT	108 bp	83
Endo16*	GACCGAACGCCGATATAAGA	GCCATCGTCCCTTTAGTTCA		
Evx	GGAGCATGCAGACATCGACAT	TGGCCACCGCTCTTAACCT	101 bp	80
FoxA*	CCAACCGACTCCGTATCATC	CGTAGCTGCTCATGCTGTGT		
FoxJ	CAAGCAGACCATGCCAAAGAA	GGGACGCAGGCTTGTAGCT	107 bp	82
FoxQ	AACGCTTCCCGTTCTTCAAAG	CTCCGCTACGTCCAGCCTT	102 bp	80
GataE	TGGCTGGCTACTCGCCATAT	CGTCCTGCAAAGTTTCCTCC	106 bp	82
Glass	GGGTACGCATGTGTGTCTTCA	TCAGGCTGCGAACCTCAAA	119 bp	80
Goosecoid*	CCCCTACACTGGCTGCCCGATGA	GCGGGCTAGTAGGTCCATGTGA		
Hairy1	CCAGAACAGGGCGAGTCAA	GATGCTCTCGCAATTGGACAT	102 bp	80
Hatching enzyme	CTGAGAATGGCGAATTTTTGC	TGTGTCGGTCCGAATGTGG	101 bp	78
Hex	ACTGCAAAGTGGGCGAGTTC	CAGCACCATAAACACGAAGCA	104 bp	82
HMX	TGCCTTTTATGCCTGGATGC	TACTGTTGGTCAGTGGCTGGG	101 bp	82
Hypothetical (crap value)	CAGCATGACTGCGCAAGGTA	TCGTTTCTGTTGCCGATGAA	102 bp	83
Iro	GCGGAGCAGGGATACGTACA	CGGCGTAACCCTCTGGAGTA	103 bp	84
KRL	TGGGAGCAAGGGTGACCTAT	GTTTCGTGGCGATGTCGAG	102 bp	82
Lefty	ACATCGACCCGATCGAGAGA	AAGTCGGTTGCGAAGACCAT	70 bp	84
Lox	TTGCAATCCAACGACCAGC	TGATGCCGTGCACCTAGATG	102 bp	81
Mox	TGAAATGGAAACGCTGCAAA	GCTAGAGCTTCCGGGTGGTA	101 bp	81
Nkx2.2	GCGTTCGCACTGGTCTCTC	TCAGGTTCCCGTTGAACACAT	144 bp	80
Nodal+	GACATCACCAAGATCGTCAAACAG	ATTCTTGTCGTGGTCAGCTTCT		

Notch*	ACGGAGCCAAGCCTAAGAA	TCGTCACAGGCAACGAATAA		
Onecut*	AACGGCTACGACCAATTCAC	GATGAGGGTGTCCCGATAGA		
OTX	AGCCACGCCCTGCTAAGAA	GCCGGGCTCCAAATACTGTT	148 bp	82
P19	CAACCCACCGAAGCTGAGAT	TTCCTGTAGAGCTGGAGCCG	101 bp	82
Pax2	GACGGGCTGGAAGAAACGT	GCTGGACAACAAGGCACTGAA	104 bp	82
PMAR1*	GCGTTCAACGACAACCAGTA	GGTTGATGAGCAGAGCTTGA		
Prox	AAGGTTACAGGGATTCGGAGC	TTGTGGTGGATACGTGCAAGA	102 bp	80
Radical spoke protein	CAACAGGCTCTGGAGACCTTG	CACACTACCAGCGACAGCTCA	125 bp	80
Six3	CCTACCGACCCTCTGCTTCTC	AGGACCACAAAAATCGGGC	101 bp	81
SM50*	CCGATTGGCCTCAACAGATCGAACCTA	GCTGTCCAGGCCCAGTGTCCACCGTCAT		
SMAD2	TCCCAAAGCTGCATCCCTT	GGTGGCTTTGAAGATCAGGC	107 bp	81
SMiP1	CAAGGAATGCGGCAAAGCT	TCTTGAGGCATCGGTCACACT	101 bp	82
Snail	CCACAGCATACGACGCAGG	CTCGGGTTTGTGGATAACTGG	101 bp	82
Sox4	CAAGTACCGTCCCCGAAAGAA	TCGATGATGACTTGGGCTTGT	101 bp	81
SoxB1*	ATTCTGTGAACGTCATGGCA	TTGTCCTCTTGACCACACCA		
SoxB2	AACATGGCATCGGCAGAGA	TTTTTGAGCGTGTTCCAGCAC	111 bp	82
SpAN	TGCTCGTTGGACCTCAAACA	GGTATGCTGCTCCCAGTGGT	103 bp	80
Spec2A*	CCACCCTTGCCACTCCTTGAAAGCAA	GCCATCGCCATCCGTGTCTTCTGATT		
Sp-sFRP1/5	TGCGCTTTCCTCGTTTCCT	ACCGGTTAACGATGGCGTATA	101 bp	82
SuH*	GCTCCATCGTTGATGATCTCT	GGACGCTGATGATCCAGTCT	156 bp	
T-Brain	AGGCACCTCTCCAAAGCTGTC	GGCGCCCTCTTGGTTGATATA	106 bp	81
Tbx2	GGGATCACCTTCGCAGACTTC	CAGAGCACCACATGTACCACG	102 bp	80
Tbx4/5	TCAGTCGGACGCAATGAGTG	CCTAGTCCGCTACCGCTCAAT	105 bp	83
Tcf/Lef	GCGAATGCTGGTTACCCAA	CCTCCCCGAGTCGTAGAGGT	103 bp	82
Ubiquitin*	CACAGGCAAGACCATCACAC	GAGAGAGTGCGACCATCCTC		
Unknown	CGTCAACCTGATCGGTTAACG	AAGCAGCATCATCAGCAGTCC	105 bp	75
WNT-A	CCGACATCATTCCAAGTGTGA	GACGTTGCTGACGGGACAGT	101 bp	83
WNT-1	ACCTTCAGAACGGTGGGTGA	AGCTTGATTCGGTTTCGCG	106 bp	83
WNT-3	TGCAAAGGCATCACCAACATA	TCGATACCGTGATAGGCGC	101 bp	82
WNT-4	AGTGGGAAGCGTTCGAAAGA	TGAGTGACGACCCTAGCGTCT	104 bp	80
WNT-5*	TGCTGTGGAAGAGGCTACAA	TTCTGCACTTCCGACACTTG		
WNT-6	CAACAGCGGCGAGAATCTC	GATCAGGCTCGCAGAAGTCG	104 bp	81
WNT-7	ACAGGATGTGCCTGCTGATTC	CATACATGCCTCGGTGATGG	103 bp	84
WNT-8*	TGTCGTTCATTCAAGCCATC	TATCACTCGCCATTCGTTCA		

WNT-9	GTTCGAAGACGGGTTGACAAG	CCGATGCTATGGAAAGGACC	101 bp	82
WNT-10	CAGTGCGGGTAAACTCCCAT	CGCCGAACTTGATGTCGTG	101 bp	83
WNT-16	CGTGAAGTCATTGCATCGGAT	CGGTTACATTCTCGTCCCGT	110 bp	80
Z12-1*	AGTCGTCCAGCCATGTCTTT	AAGCACACCTCGCACCTATC		
ZFhpf4	ACCCCACCGTTGTCATGAAT	CGCAGAACTCGCATATGAATG	107 bp	81

* from http://sugp.caltech.edu/resources/methods/q-pcr.php

+ from **Flowers, V. L., Courteau, G. R., Poustka, A. J., Weng, W. and Venuti, J. M.** (2004). Nodal/activin signaling establishes oral-aboral polarity in the early sea urchin embryo. *Dev Dyn* **231**, 727-40.
